# LATS1/2 inactivation drives a distinct venous endothelial cell response that contributes to fibrotic remodeling of the lung

**DOI:** 10.1126/sciadv.aef8468

**Published:** 2026-08-28

**Authors:** Konstantinos Kontodimas, Ahmed A. Raslan, Benjamin Spira, Crystal Zhu, Janardhan Karapurkar, Yohana Kefella, Darren Chiu, Xintao Qiu, Jennifer E. Beane, Giovanni Ligresti, Xaralabos Varelas

**Affiliations:** ^1^ Department of Biochemistry and Cell Biology, Boston University Chobanian and Avedisian School of Medicine, Boston, MA, USA.; ^2^ Pulmonary Center, Boston University, Boston, MA, USA.; ^3^ Department of Medicine, Boston University Chobanian and Avedisian School of Medicine, Boston, MA, USA.; ^4^ Arthritis and Autoimmune Diseases Center, Boston University Chobanian and Avedisian School of Medicine, Boston, MA, USA.; ^5^ Bioinformatics Program, Boston University, Boston, MA, USA.

## Abstract

Endothelial dysfunction is recognized to contribute to chronic tissue remodeling in the lung, yet endothelial-derived mechanisms driving these processes remain largely undefined. Here, we show that endothelial inactivation of the LATS1 and LATS2 kinases, key suppressors of the transcriptional regulators YAP and TAZ, elicits distinct responses depending on endothelial identity within different pulmonary vascular beds. Our data indicate that LATS1/2 inactivation in general capillary endothelial cells induces a reactive capillary injury-like state, whereas a separate endothelial population located within veins adopts a distinct profibrotic state. We show that expansion of this reactive venous endothelial cell population, which is marked by EBF1 and ACKR1 expression, is associated with fibroblast activation, macrophage accumulation, and epithelial remodeling, collectively generating a microenvironment characteristic of fibrotic lung disease. We further demonstrate that pharmacologic inhibition of YAP/TAZ-TEAD signaling prevents stromal and immune remodeling and fibrotic lesion formation following endothelial LATS1/2 inactivation. These findings identify LATS1/2-mediated restraint of YAP/TAZ as essential for lung endothelial homeostasis and highlight a distinct venous cell response as a direct contributor to fibrotic lung remodeling.

## INTRODUCTION

The pulmonary endothelium forms an extensive and specialized cellular network that is essential for maintaining lung structure and function, serving as a barrier that regulates gas exchange, fluid balance, and immune surveillance at the blood-air interface (*1*). Lung endothelial cells (ECs) are heterogeneous and adapted to perform distinct functions reflecting their location within the vasculature. Capillary ECs are prevalent throughout lung alveoli and can be divided into specialized subpopulations that include aerocytes (aCap ECs, also known as CAP2 ECs) that mediate gas exchange and general capillary ECs (gCap ECs, also known as CAP1 ECs) that support vascular homeostasis (*2*–*4*). These capillary ECs connect through precapillary arterioles and postcapillary venules to pulmonary arteries and veins, respectively, each of which exhibit distinct characteristics that direct vascular tone, hemodynamic regulation, and oxygenation (*5*). Functioning as an early site of injury, responses that are mediated by lung EC populations are emerging as key contributors to lung regeneration (*6*–*9*), age-related lung disfunction, and to the pathogenic states of lung diseases (*10*–*15*). The heterogeneous dynamics of EC to injury and disease are only starting to be understood, and data are emerging suggesting distinct contributions to remodeling of the local microenvironment.

Lung EC dysfunction is notably associated with pulmonary hypertension, which involves maladaptive remodeling of ECs, smooth muscle expansion, and perivascular inflammation (*16*). Such cellular changes in pulmonary hypertension collectively contribute to the occlusion of pulmonary arteries or more rarely of the veins that ultimately culminates in heart failure. Pulmonary fibrosis, a chronic, progressive lung disease characterized by excessive deposition of extracellular matrix and scarring of the lung parenchyma (*17*), has also been increasingly linked to lung endothelial dysfunction. Recent studies (*14*, *15*, *18*–*20*), including our own (*10*, *21*), have revealed altered cell states that associate with fibrotic responses post–lung injury, with distinct EC states that are spatially localized near collagen-rich fibrotic foci. Fibrosis-associated EC states exhibit transcriptional programs linked to inflammatory, hypoxic, and metabolic changes, suggesting that they may directly contribute to the fibrotic program. For example, lineage tracing in mice following bleomycin-induced injury demonstrate that the Apelin Receptor (Aplnr)–expressing ECs, primarily gCap ECs, undergo cell states transitions that closely associate with fibrotic collagen remodeling, with data suggesting a role for the transcriptional effectors YAP and TAZ as drivers of this response (*10*).

YAP and TAZ function as major mechanotransducing factors that control lung cell growth, differentiation, and fibrosis (*22*–*29*) and have emerged as key regulators of vascular development and remodeling (*30*–*33*). YAP and TAZ are transcriptional effectors of the Hippo signaling pathway, functioning to regulate diverse gene expression programs through transcription factor networks that include the TEAD family of transcription factors (*24*). A primary mechanism of YAP/TAZ regulation is phosphorylation by the LATS1 and LATS2 (LATS1/2) kinases, which direct the phosphorylation of YAP/TAZ on conserved residues, resulting in restricted YAP/TAZ nuclear accumulation and hence inhibited transcriptional activity (*34*). LATS1/2 and YAP/TAZ activity is regulated by numerous upstream signals, many of which are transduced through cellular cytoskeletal changes, notably including those regulated by mechanical forces (*35*). YAP/TAZ nuclear activity is transiently activated with tissue injury, and persistent nuclear YAP/TAZ activity is often observed in diseases with altered tissue mechanics, including with the extracellular matrix stiffening linked to pulmonary fibrosis (*25*).

To test the consequences of persistent activation of YAP/TAZ in the pulmonary endothelium, we developed a genetic mouse model enabling the conditional deletion of the *Lats1* and *Lats2* genes in Aplnr-expressing cells. We achieved this by using the tamoxifen-inducible Aplnr-CreERT2 model, which is known to target the lung microvascular gCap ECs and some ECs in postcapillary arteriolar and venule endothelium in adult mice (*10*, *36*). We found that sustained Lats1/2 inactivation in Aplnr-expressing endothelium results in the formation of widespread foci of increased cellularity (lesions) throughout the lungs that associate with stromal remodeling, collagen deposition, and immune cell recruitment. Single-cell RNA sequencing (scRNA-seq) coupled with histology identified two EC lineages that were sensitive to Lats1/2 inactivation, including the expansion of a distinct EC population within the venous vascular bed that is marked by unique factors (such as Ebf1 and Ackr1), and a capillary-like population that is marked by factors often observed with lung injury (such as Cxcr4). Lats1/2 inactivation in Aplnr-expressing ECs is sufficient to drive broad non-EC changes throughout the lungs, including responses by fibroblast, monocyte, and macrophage populations that are linked to fibrotic lung remodeling, along with the induction of inflammatory-associated alveolar epithelial transitional states, all of which parallel observations in chronic injury–associated diseases, such as pulmonary fibrosis. Our data suggest that many of these phenotypes, including elevated collagen deposition, are coupled to the distinct venous-like cells that emerge following Lats1/2 inactivation, suggesting a key role for these cells in establishing a fibrotic niche. We further found that blocking nuclear YAP/TAZ activity with an inhibitor of the TEAD transcription factor family reduced the emergence of fibrotic lesions in Lats1/2 deficient lungs, implicating YAP/TAZ-TEAD as central factors in the observed phenotypes. Collectively, these data implicate the distinct subsets of pulmonary ECs as direct contributors to the onset of fibrotic lung dysfunction and identify the Lats1/2-YAP/TAZ signal axis as a central mediator of maladaptive EC state transitions, opening the door to potential strategies for targeting EC dysfunction in the lung.

## RESULTS

### LATS1/2 deletion in Aplnr-expressing lung ECs drives the expansion of fibrotic venous lesions

**Fig. 1.**
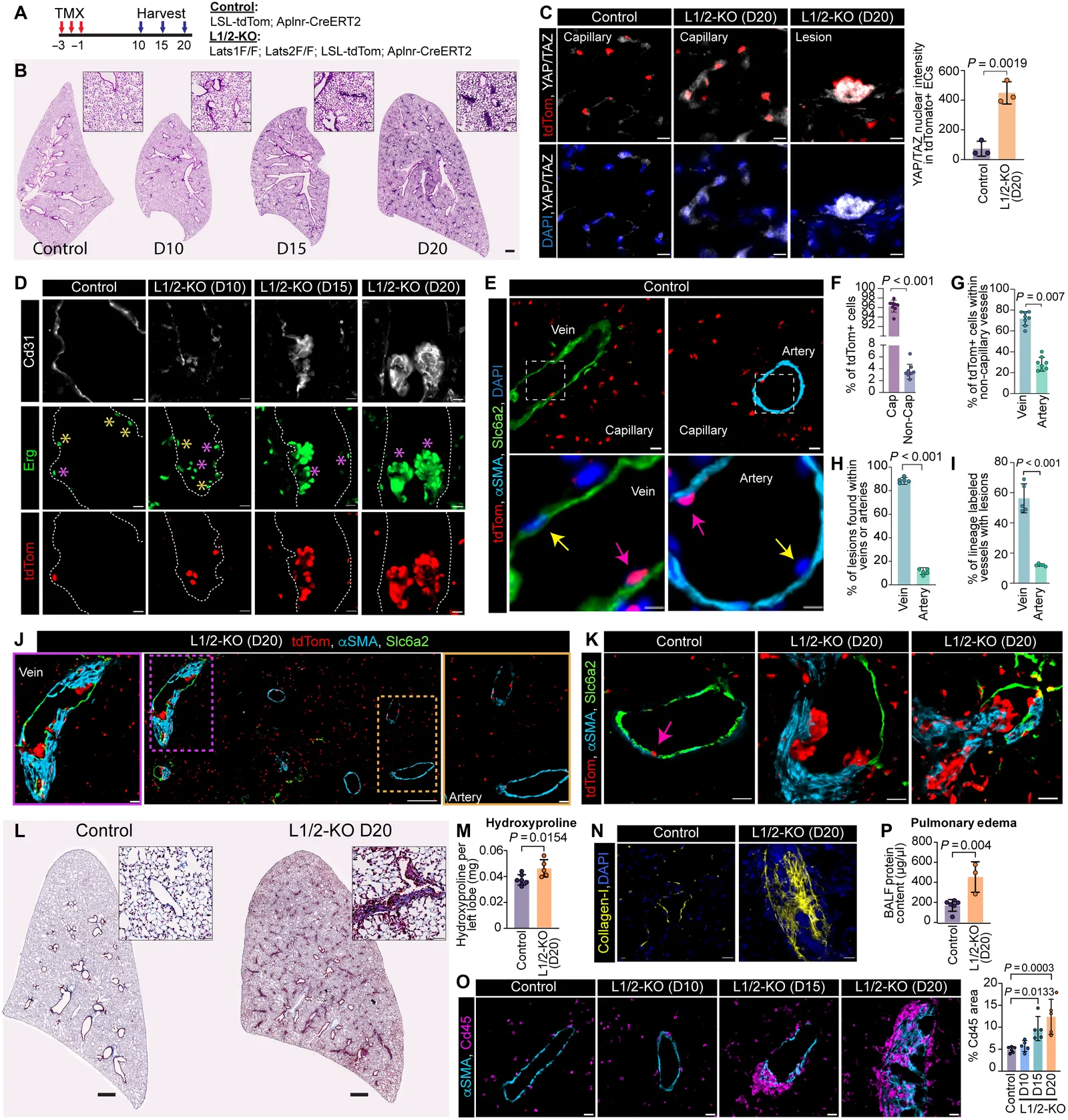
Lats1/2 inactivation drives expansion of distinct lung venous ECs associated with cellular remodeling and aberrant collagen deposition. (**A**) Study design schematic. TMX, tamoxifen. (**B**) H&E staining of control and Lats1/2-KO lungs at day 10 (D10), day 15 (D15), and day 20 (D20) post-tamoxifen [scale bars, 500 μm and 100 μm (insets). (**C**) IF for tdTom and YAP/TAZ in control and Lats1/2-KO D20 lungs (scale bars, 10 μm) with quantification of nuclear YAP/TAZ in tdTom+ cells (*n* = 3 per group). (**D**) IF for Cd31, tdTom, and Erg (scale bars, 20 μm); asterisks denote tdTom+ (purple) or non-tdTom ECs (yellow). (**E**) IF for tdTom, αSMA, and Slc6a2 in control lungs (top: 10 μm; bottom: 5 μm); purple arrows indicate tdTom+ ECs, and yellow arrows indicate non-tdTom ECs within veins (left) or arteries (right). (**F**) Quantification of the tdTom+ EC distribution in capillary versus noncapillary beds (*n* = 8). (**G**) Quantification of tdTom+ ECs in venous versus arterial vessels (*n* = 8), (**H**) lesion localization (*n* = 5), and (**I**) proportion of arterial and venous vessels with lesions (*n* = 5). (**J**) IF for tdTom, αSMA, and Slc6a2 in Lats1/2-KO D20 lungs (middle: 100 μm; sides 20 μm). (**K**) IF comparison of control and Lats1/2-KO D20 lungs (scale bars, 20 μm). (**L**) Masson’s trichrome staining of control and Lats1/2-KO lungs [scale bars, 500 μm and 100 μm (insets)]. (**M**) Hydroxyproline quantification (*n* = 7 control; *n* = 5 KO). (**N**) IF for collagen I (scale bars, 20 μm). (**O**) IF for Cd45 and αSMA on control and Lats1/2-KO D10, D15, and D20 lungs (scale bars, 20 μm) with quantification of Cd45+ area (*n* = 6 control; *n* = 5 KO/time point). (**P**) Protein levels in BALF (*n* = 6 control; *n* = 3 KO). All values are means ± SEM. A one-way analysis of variance (ANOVA) was used for (O), and two-tailed Student’s *t* test was used for (C), (F) to (I), (M), and (P).

To investigate the consequences of sustained nuclear activation of the transcriptional regulators YAP and TAZ in lung capillary ECs, we crossed *Aplnr*-CreERT2 mice (*37*) with *Lats1*-floxed and *Lats2*-floxed mice (*38*, *39*) and incorporated a lox-stop-lox-tdTomato lineage tracing reporter (*40*) to track targeted cells following tamoxifen administration (Aplnr-Lats1/2-cKO^tdtom^) (Fig. 1A). In addition to the use of Cre negative mice as controls, *Aplnr*-CreERT2 mice crossed with the lox-stop-lox-tdTomato reporter (Aplnr-CTL^tdtom^) were also generated as lineage tracing controls. Upon tamoxifen administration we found that Aplnr-Lats1/2-cKO^tdtom^ animals develop widespread areas of increased focal cellularity (herein called lesions) throughout their lungs that progress in size over time (Fig. 1B). Immunofluorescence (IF) staining revealed elevated levels of YAP and TAZ (YAP/TAZ) throughout tdTomato+ cells found within lesions that develop in Aplnr-Lats1/2-cKO^tdtom^ lungs compared to controls (Fig. 1C), suggesting a role for YAP/TAZ in lesion development. We found that cells within these lesions retain the expression of Cd31 and the Erg transcription factor (Fig. 1D), indicating the maintenance of endothelial identity (*41*). We also observed that some cells within the lesions exhibited a proliferative response, which was marked by 5-ethynyl-2′-deoxyuridine (EdU) incorporation and staining (fig. S1A).

Unexpectedly, we noticed that, although ECs within the capillary space exhibited efficient tdTomato lineage labeling and elevated YAP/TAZ levels (Fig. 1C), lineage-labeled lesions in Aplnr-Lats1/2-cKO^tdtom^ lungs appeared to expand in areas resembling larger vascular vessels (Fig. 1D). This observation prompted us to carefully investigate the cells being targeted by the Aplnr-CreERT2 model. For this, we examined tdTomato labeling within the Aplnr-CTL^tdtom^ controls comparing labeling within the capillary bed and noncapillary bed, distinguishing veins by costaining for the marker Slc6a2, recently shown to be expressed in venous ECs (*42*), and α–smooth muscle actin (αSMA), which composes the thick band of smooth muscle cells (SMCs) (αSMA^high^) that surrounds arterial vessels. All tdTomato-expressing cells that were detected coexpressed Erg, indicating that the Aplnr-CreERT2 lineage trace adhered to ECs. The large majority of these lineage traced ECs (∼96%) were found within the capillary bed, consistent with efficient labeling of gCap ECs (*3*) (Fig. 1, E and F). We also observed small numbers (∼4%) of Aplnr lineage–traced cells localized within veins (Slc6a2^+ve^/αSMA^low^) and arteries (Slc6a2^-ve^/αSMA^high^) of various sizes (Fig. 1, E and F, and fig. S1B), with more observed within veins (Fig. 1G), coexisting with nonlabeled cells within the same vessel, suggesting heterogeneous EC composition within the same vascular bed.

We extended our analysis to the Aplnr-Lats1/2-cKO^tdtom^ lungs, focusing on venous (Slc6a2^+ve^/αSMA^low^) and arterial (Slc6a2^-ve^/αSMA^high^) vessels. This analysis demonstrated that tdTom+ lesions primarily (∼90% of all lesions) arose in Slc6a2+ venous vessels (Fig. 1, H and I) and that about 50% of all marked veins exhibited lesion formation, compared to 10% of arteries (Fig. 1J), suggesting a distinct venous EC response to Lats1/2 deletion. tdTomato+ cells within Aplnr-Lats1/2-cKO^tdtom^ lesions exhibited reduced Slc6a2 levels and were observed to expand intraluminally or outward into the neighboring lung parenchyma (Fig. 1K). We also noticed that cell aggregates within lesions exhibited a notable accumulation αSMA+ cells (Fig. 1, J and K, and fig. S1C), leading to large areas of αSMA+ coverage that resemble myofibroblast accumulation that is often associated with tissue injury and fibrosis. These observations prompted us to examine collagen levels, which showed higher levels of collagen accumulating in lesion areas developing in Aplnr-Lats1/2-cKO^tdtom^ lungs, measured by Masson’s trichrome (Fig. 1L) and Picrosirius Red staining (fig. S1D) and further quantified with whole-lung hydroxyproline measurements (Fig. 1M). Collagen type 1 levels were particularly elevated in these developing lesions (Fig. 1N and fig. S1E). Given the high cellularity of the lesions, we also stained for Cd45+ immune cells, which revealed progressive immune accumulation at Lats1/2-cKO venous-associated lesion sites (Fig. 1O).

Aplnr-Lats1/2-cKO^tdtom^ lungs also exhibited elevated protein content in bronchoalveolar lavage fluid (BALF), a measure of alveolar-capillary barrier breakdown and pulmonary edema (Fig. 1P). The possibility of endothelial barrier defects were therefore examined through Evans Blue vascular permeability assays, which demonstrated that Aplnr-Lats1/2-cKO^tdtom^ lungs exhibit defective barrier capacity compared to controls, resulting in enhanced lung vascular leak (fig. S1F). To test the direct impact of Lats1/2 depletion on lung EC junctional integrity (versus potential indirect effects that could result from inflammation), we examined human lung microvascular ECs treated with control small interfering RNA (siRNA) or siRNA targeting Lats1/2. Lats1/2-depleted cells showed marked inhibition of EC junction formation (fig. S1G), compromised transendothelial electrical resistance (TEER) (fig. S1H), and enhanced the permeability of fluorescent dextran dye from the apical-to-basal culture chambers (fig. S1I). These data indicate that LATS1/2 are crucial for homeostatic maintenance of lung EC polarity, similar to that observed in other cell types (*43*–*45*), including ECs in other contexts (*30*, *46*, *47*).

### Aplnr-expressing lung ECs exhibit a heterogeneous response to Lats1/2 deletion

**Fig. 2.**
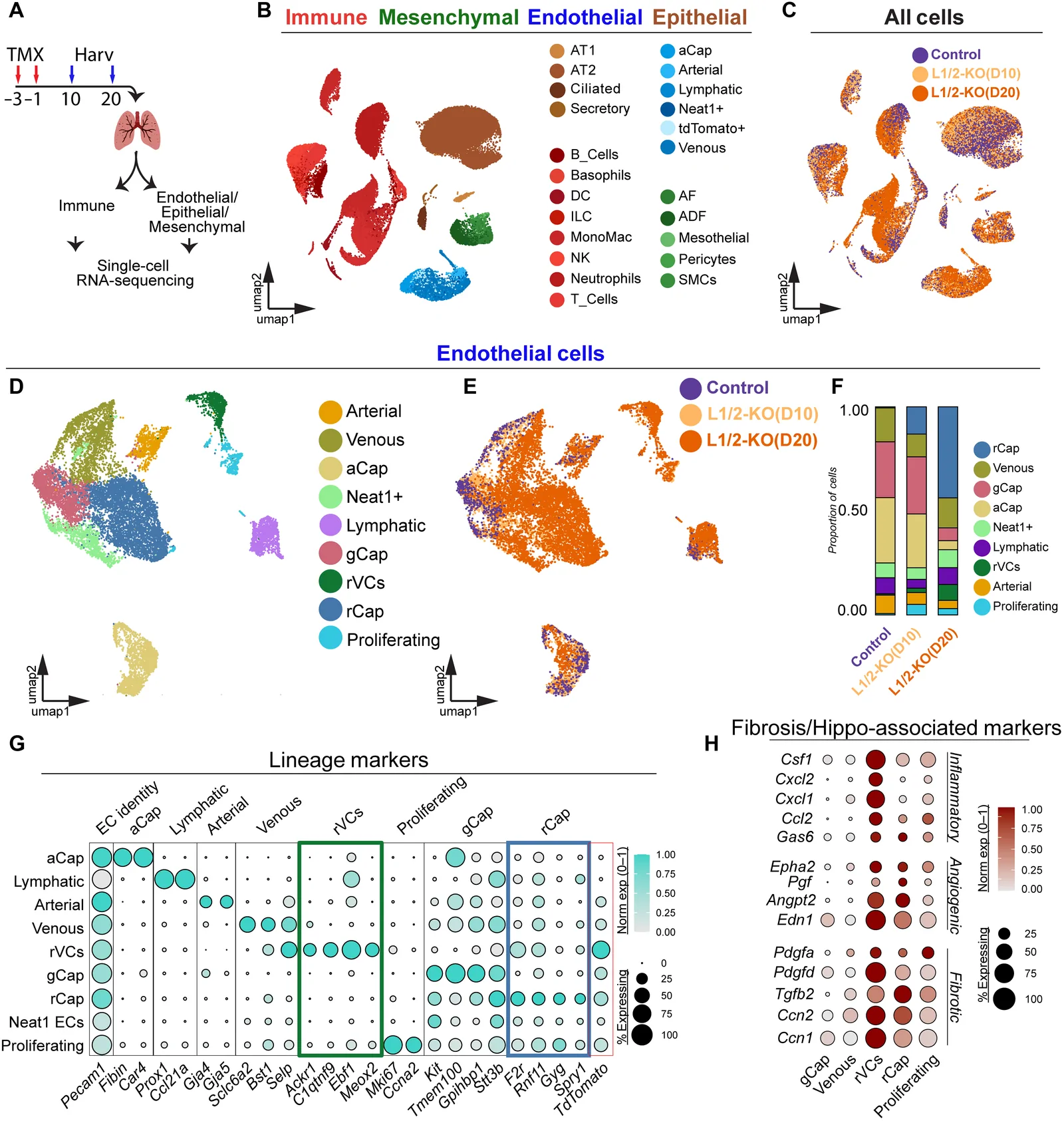
Lats1/2 inactivation using the Aplnr-CreERT2 model leads to marked lung cellular remodeling, identified by scRNA-seq, including the emergence of two distinct EC populations. (**A**) Schematic of the scRNA-seq strategy to analyze control and Lats1/2-KO lungs [*n* = 4 mice for control, 3 mice for Lats1/2-KO(D10), and 2 mice for Lats1/2-KO(D20)]. (**B**) UMAP of all captured cells clustered on the basis of their cell lineage (red: immune; light blue: endothelial; brown: epithelial; green: mesenchymal) (*n* = 135,193 cells). (**C**) UMAP of all captured cells with each condition and time point highlighted [purple: control; light brown: Lats1/2-KO(D10); orange: Lats1/2-KO(D20)]. (**D**) UMAP of unbiased subclustering of ECs (*n* = 14,289 cells). (**E**) UMAP of subclustered ECs highlighting each condition and time point. (**F**) Proportion analysis of EC populations identified across conditions and time points. (**G**) Bubble plot highlighting distinct EC lineage markers. (**H**) Bubble plot highlighting inflammatory/angiogenic, fibrosis, and Hippo pathway-related markers.

To gain insight into the cellular complexity arising in Aplnr-Lats1/2-cKO^tdtom^ lungs, we used scRNA-seq to examine early (day 10) and late (day 20) time points post–tamoxifen treatment (Fig. 2A), comparing to Aplnr-CTL^tdtom^ control lungs treated with tamoxifen (control). Lungs were harvested and processed into single-cell suspensions that were enriched as pools of immune (Cd45+) or Cd45-negative mesenchymal cells, epithelial cells, and ECs and then sequenced using the 10x Genomics platform. After quality filtering, we obtained 135,193 cells, which were divided into 42,513 control cells, 37,825 day 10 cells, and 54,855 day 20 cells and examined by clustering in UMAP space (Fig. 2, B and C). We successfully captured all major lung lineages and respective subpopulations (Fig. 2, B and C), which are marked by canonical markers of endothelium (*Cdh5*), immune (*Ptprc*), mesenchymal (*Col1a1*), and epithelium (*Nkx2.1*) (fig. S2A).

As a start to profiling the scRNA-seq data, we subclustered all *Cdh5/Pecam1* expressing cells (14,289 cells) to examine responses in ECs. Unbiased clustering revealed nine major endothelial subpopulations, which included Venous ECs, Lymphatic ECs, Arterial ECs, gCap ECs, aCap ECs, and a capillary-like population marked by the expression of *Neat1*. Three EC populations were distinctly expanded in the Lats1/2-cKO samples (Fig. 2, D to F), including one with capillary-like markers that we named reactive capillary (rCap) ECs, one with venous-like markers that we named reactive venous cells (rVCs) and one with markers of proliferation (Fig. 2G). Clear populations shifts were observed following Lats1/2 deletion, including a progressive decrease in the gCap ECs with corresponding increases in the rCap and rVC ECs (Fig. 2F). The proliferative population showed an early increase that dissipated at the later time point (Fig. 2F). Analysis for transcripts of the tdTomato lineage trace revealed expression in ECs (fig. S2B), primarily in four major populations that included gCap ECs, which represented most tdTomato+ cells in controls, and the rCap ECs, rVCs, and proliferating ECs, which were progressively increased in the Aplnr-Lats1/2-cKO^tdtom^ lungs (fig. S2, C and D). The cell clusters associated with the Aplnr-Lats1/2-cKO^tdtom^ lungs showed distinct induction expression patterns for previously recognized YAP/TAZ target genes, including profibrotic (*Ccn1*, *Ccn2*, *Tgfb2*, *Pdgfa*, and *Pdgfb*), immunomodulatory functions (*Csf1*, *Cxcl1*, *Cxcl2*, *Ccl2*, and *Gas6*), and angiogenic factors (*Edn1*, *Pgf*, *EphA2*, and *Angpt2*) (Fig. 2H and table S1). These data suggest that endothelial Lats1/2 inactivation affects distinct transcriptional programs in different EC populations that likely reflect their specialization within the pulmonary vasculature.

### Lats1/2 deletion in Aplnr-expressing lung ECs drives the expansion of a distinct venous-associated endothelial population that associates with lung scarring

**Fig. 3.**
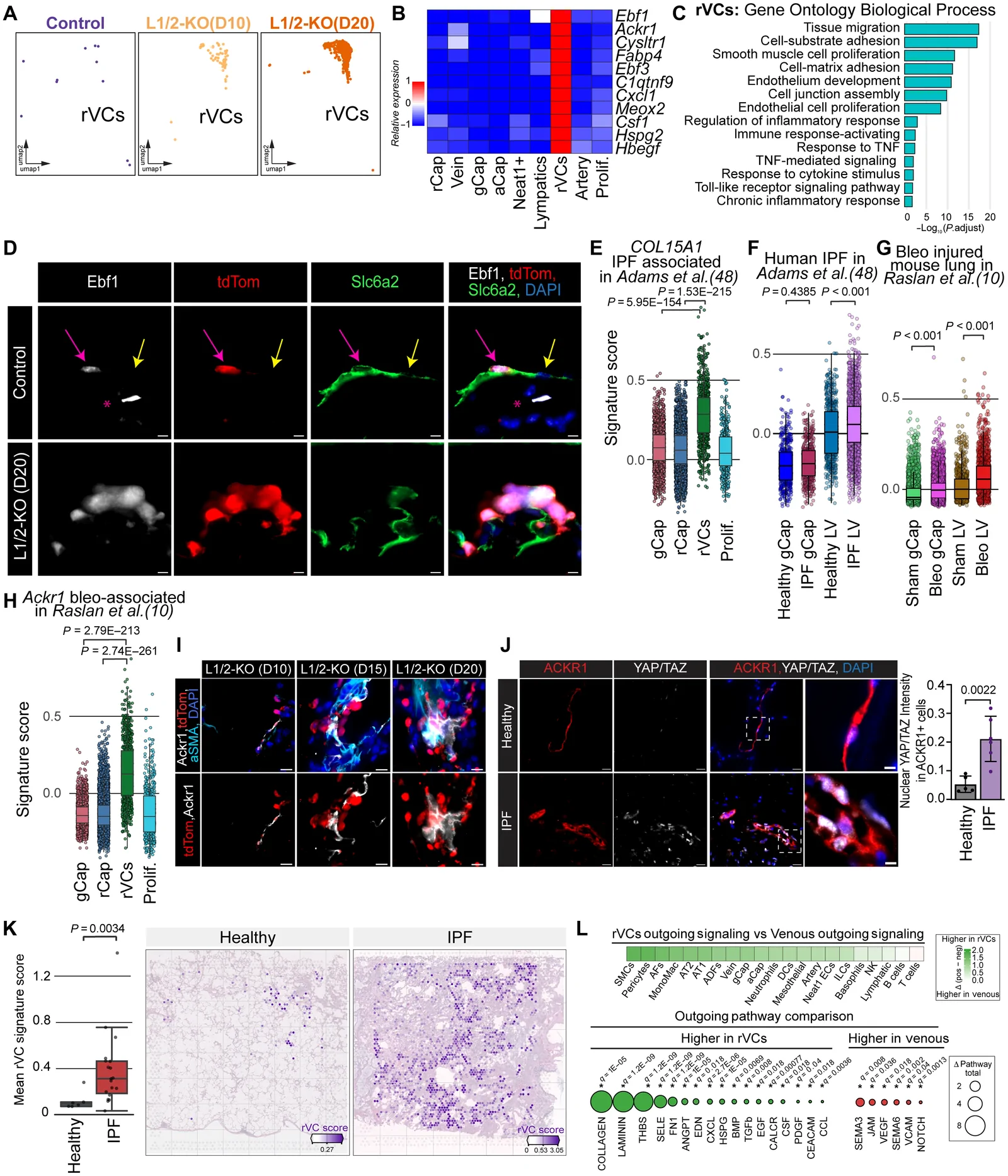
Lats1/2 inactivation expands distinct venous ECs associated with pulmonary fibrosis. (**A**) UMAP of all rVCs from our scRNA-seq, colored by condition and time. (**B**) Heatmap of genes enriched in rVCs. (**C**) GO analysis of rVC genes. (**D**) IF for tdTom, Ebf1, and Slc6a2 in control and Lats1/2-KO D20 lungs (scale bars, 5 μm); arrows denote tdTom+ rVCs with Ebf1 (pink) and non-tdTom ECs without Ebf1 (yellow); neighboring Ebf1+ mesenchymal cells marked with pink asterisks. (**E**) Enrichment of the human COL15A1 vein gene signature [Adams *et al.* (*48*)] across tdTom+ cells. (**F** and **G**) Enrichment of the rVC signature in (F) human IPF ECs [Adams *et al.* (*48*)] or (G) mouse bleomycin fibrosis ECs [Raslan *et al.* (*10*)]. (**H**) Enrichment of mouse Ackr1+ EC signature [Raslan *et al.* (*10*)] across tdTom+ cells. Values are summarized as boxplots (interquartile range) with jittered single-cell points. (**I**) IF for tdTom, αSMA, and Ackr1 in Lats1/2-KO lungs (scale bars, 20 μm). (**J**) IF for ACKR1 and YAP/TAZ in human healthy and IPF lungs (20 μm; inset, 5 μm) with quantification of nuclear YAP/TAZ in ACKR1+ ECs (*n* = 5 healthy; *n* = 6 IPF). (**K**) Enrichment and spatial mapping of the rVC gene signature in human lung spatial transcriptomics data [Franzén *et al.* (*90*)]. (**L**) CellChat analysis of outgoing signaling changes per receiver between rVCs and Venous ECs, with relative change in interaction strength toward recipient (top) and differential outgoing signaling per sender toward all receivers (bottom). Delta/circle size signifies relative change in pathway-level communication probability difference between rVC and Venous ECs. Statistical analysis: [(E) and (H)] nonparametric two-sided Kruskal-Wallis rank sum tests, followed by Dunn’s post hoc test with Benjamini-Hochberg FDR correction; [(F) and (G)] nonparametric two-way aligned rank transform ANOVA followed by Benjamini-Hochberg–adjusted post hoc contrasts; (J) two-tailed Student’s *t* test; (K) nonparametric Wilcoxon rank sum test; (L) two-sided Wilcoxon rank sum tests, followed by Benjamini-Hochberg FDR correction across pathways.

Venous ECs have been recently demonstrated to expand in human and mouse fibrotic lungs (*10*, *48*), and thus our observations of an rVC population following Lats1/2 deletion warranted further investigation. rVCs were found to represent 0.6% of ECs in control lungs, indicating the presence of such cells at adult lung homeostasis, and upon Lats1/2 deletion were found to progressively accumulate (day 10 with 1.98% and day 20 7.6% of ECs) (Fig. 3A). rVCs clustered closely with the proliferating EC group (Fig. 2D), suggesting a proliferative expansion of this population following Lats1/2 inactivation. Genes marking rVCs included those encoding transcription factors (*Ebf1*, *Ebf3*, and *Meox2*) and immune recruiting factors (*Csf1*, *Cysltr1*, and *Cxcl1*) (Fig. 3B and table S2). Gene set enrichment analysis for biological processes revealed enrichment for cell-substrate adhesion, regulation of blood vessel EC migration, and inflammatory signaling (Fig. 3C), suggesting a promigratory/inflammatory state arising from Lats1/2 inactivation.

To further test and validate the relationship between the rVC transcriptomic program and the venous-associated lesions observed in the Aplnr-Lats1/2-cKO^tdtom^ lungs, we stained for Ebf1. We found that Ebf1 marks the expanding tdTomato+ cells observed within Aplnr-Lats1/2-cKO^tdtom^ lesions (Fig. 3D). We observed that tdTomato+ cells within Aplnr-CTL^tdtom^ control lungs also express Ebf1 in a discrete pattern to adjacent ECs within venous vessels (Fig. 3D), suggesting that presence of rVCs at homeostasis. Accordingly, *Ebf1* expression marks rVCs that were identified in our scRNA-seq experiment (fig. S3A). We also found that *Ebf1* is expressed in subsets of mesenchymal cells, as previously reported (*13*, *49*). Notably, a prior work has reported the expansion of a *COL15A1*-expressing venous-like EC population in human idiopathic pulmonary fibrosis (IPF) that expresses markers resembling the rVC population that expands with Lats1/2 inactivation, including *EBF1*, *MEOX2*, and *CYSLTR1* (*4*). This prompted us to evaluate the similarities between rVCs and the *COL15A1* population identified in human IPF. For this, we generated a gene signature for the *COL15A1* EC population in human IPF using highly expressed unique genes identified in those cells (table S3) and found strong enrichment in the rVCs identified in the Aplnr-Lats1/2-cKO^tdtom^ lungs, an enrichment that does not exist with other EC populations in this model (Fig. 3E). The converse analysis of examining highly expressed genes in rVCs within human IPF data also showed enrichment in IPF-associated large vessel ECs (Fig. 3F), supporting the similarity in these cell populations with those observed to expand in human IPF. To further explore the presence of rVCs in human lungs and association with IPF we examined another human IPF scRNA-seq dataset that efficiently captured ECs (*50*). Similarly, we found an enrichment of the rVC gene signature in ECs of large vessels found in human IPF (fig. S3B). This dataset captured ECs from both healthy and IPF lungs, with transcriptomic differences observed across the different EC populations (fig. S3, C and D). We interrogated this dataset to explore markers of rVCs and found that healthy human lungs have a small population of ECs that express *EBF1*, *MEOX2*, *ACKR1*, and *COL15A1* (fig. S3E), supporting the existence of rVCs in human lungs at homeostasis. Furthermore, cells expressing these markers expand in human IPF lungs, suggesting a distinct response under disease conditions. We have also similarly observed the expansion of venous ECs with markers that include Ackr1 in the mouse bleomycin-induced fibrosis model and in human IPF lungs (*10*). We found that the rVC gene signature was enriched in the Ackr1-expressing venous population (Fig. 3G) and accordingly, large vessel ECs in fibrotic lungs from old mice were enriched for the rVC gene signature (Fig. 3H). Consistent with these similarities, we found that Ackr1 protein was present in the venous-associated tdTomato+ aggregates observed in Aplnr-Lats1/2-cKO^tdtom^ lungs (Fig. 3I). Examination of human healthy and IPF lung tissues demonstrated that ACRK1-expressing cells exhibit elevated YAP/TAZ levels, with notable increases in the nuclear levels (Fig. 3J), consistent with a functional role for YAP/TAZ in ECs in IPF. We extended our analysis to explore the spatial profile of the rVC gene signature to recent spatial transcriptomics data from human healthy and IPF lung tissues. We observed enrichment of rVC gene scores in aggregated areas that were elevated in IPF compared to healthy lungs (Fig. 3K), resembling patterns reminiscent of the rVC lesions in Aplnr-Lats1/2-cKO^tdtom^ lungs.

To gain initial insight into potential paracrine signals that are induced within rVCs examined our scRNA-seq data using CellChat, a computational tool that integrates expression of ligands, receptors, and cofactors using prior knowledge of signaling patterns to predict cross-talk between cell groups (*51*, *52*). We focused on outgoing signals derived from rVCs and compared these to control venous cells, which revealed elevated signaling within rVCs primarily to SMCs, pericytes, fibroblasts, monocytes, and macrophages (Fig. 3L, fig. S3F, and table S4). These outgoing signals included general increases in extracellular matrix–mediated signaling (such as Collagen, Laminin, Thrombospondin, Fibronectin, and HSPG), growth factor signaling (TGFβ, BMP, EGF, PDGF, EDN, and ANGPT), and immune recruitment/regulation (CSF, CXCL, CCL, MIF, and Selectin). Several signaling events were also reduced in rVCs, which notably included Semaphorin signaling.

### Lats1/2 deletion in Aplnr-expressing lung ECs drives the induction of a distinct capillary EC state

**Fig. 4.**
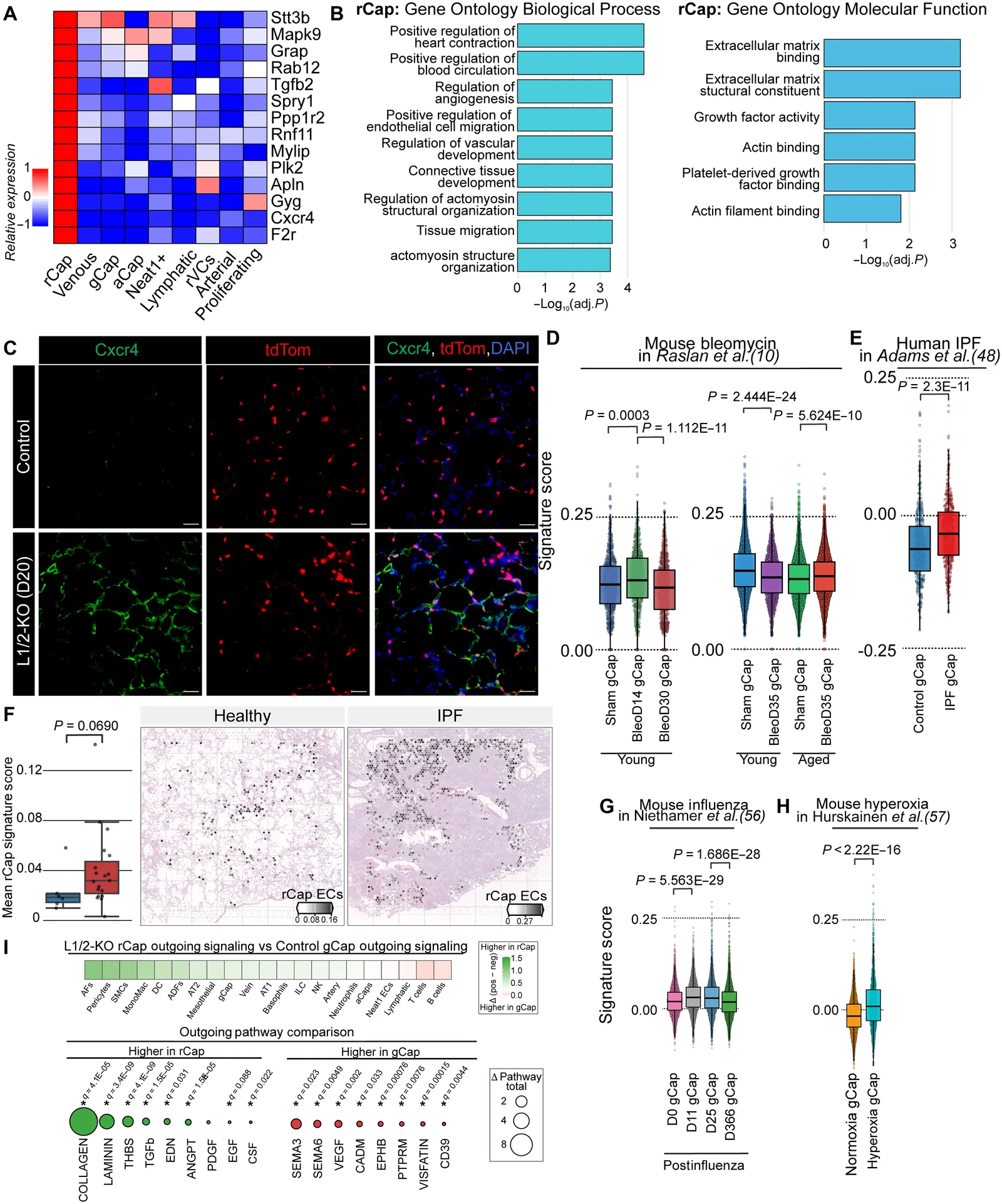
Lats1/2 inactivation in Aplnr-expressing capillary ECs drives a distinct gene expression program that resembles gCap ECs following lung injury. (**A**) Heatmap highlighting genes enriched in rCap ECs induced by Lats1/2-KO. (**B**) GO enrichment of genes distinctly expressed in rCap ECs. (**C**) IF for Cxcr4 and tdTom on control and Lats1/2-KO D20 lungs (scale bars, 20 μm). (**D** and **E**) rCap EC signature score enrichment on (D) bleomycin-treated mouse gCap ECs [Raslan *et al.* (*10*)] and (E) human IPF gCap ECs [Adams *et al.* (*48*)]. (**F**) Enrichment of the Lats1/2-KO rCap gene signature across healthy and IPF samples from the Franzén *et al.* dataset (*90*) (left). Spatial mapping of the Lats1/2-KO-rCap gene signature across a healthy and IPF sample (right). (**G** and **H**) rCap EC signature score enrichment on gCap ECs from (G) influenza-injured [Niethamer *et al.* (*56*)] and (H) hyperoxia-injured [Hurskainen *et al.* (*57*)] lungs. (**I**) Heatmap depicting CellChat analysis comparing the total outgoing signaling strength per receiver between Lats1/2-KO (D10 and D20 combined) rCap and control gCap ECs. Delta/color signifies relative change in interaction strength toward each recipient (top). Bubble plot depicting differential outgoing signaling per sender, by summing pathway-level communication probabilities from Lats1/2-KO rCap and control gCap ECs (bottom). Delta/circle size signifies relative change in pathway-level communication probability difference between rCap and gCap ECs. For (F), values are summarized as boxplots (interquartile range) with jittered single-cell points and a nonparametric Wilcoxon rank sum test was performed. For (D), (E), (G), and (H), values are summarized as boxplots (interquartile range) with jittered single-cell points. Significance was assessed using two-sided Wilcoxon rank sum tests for (E) and (H). A nonparametric two-sided Kruskal-Wallis rank sum test, followed by Dunn’s post hoc test with Benjamini-Hochberg FDR correction was used for [(D), left] and (G). A nonparametric two-way aligned rank transform ANOVA followed by Benjamini-Hochberg–adjusted post hoc contrasts was used for [(D), right].

The most prominent EC shift identified by scRNA-seq in Aplnr-Lats1/2-cKO^tdtom^ lungs compared to controls was a population we termed rCap ECs, for their transcriptional resemblance to capillary ECs. The primary EC population targeted by the Aplnr-CreERT2 models are gCap ECs (*3*, *10*), suggesting that rCap ECs may arise from Lats1/2 deletion–mediated plasticity of gCap ECs. Distinct genes were found to be enriched in rCap ECs (Fig. 4A and table S5) with Gene Ontology (GO) analysis indicating roles in vascular development, cell migration, actin remodeling, and angiogenesis (Fig. 4B). To investigate the location of these cells we stained for Cxcr4, which we found was elevated in the capillary bed of Aplnr-Lats1/2-cKO^tdtom^ lungs (Fig. 4C), but not in the venous-associated lesions (fig. S4A). Cxcr4 is a receptor for Cxcl12 (also known as stromal cell–derived factor 1, Sdf1) that is known to respond to lung injury and facilitates EC movement, barrier dysfunction, and immune cell recruitment (*53*–*55*). Cxcl12, which is normally secreted by gCap and arterial ECs, was found to be highly induced in the rVC population (fig. S4B), suggesting potential new communication arising between these cell populations in Aplnr-Lats1/2-cKO^tdtom^ lungs.

To gain insight into the meaningfulness of the rCap EC population, we investigated genes distinctly expressed in this population across different lung injury datasets (table S3). We found that rCap EC genes were enriched in gCap ECs observed in bleomycin injured lungs of old mice that show persistent fibrosis (Fig. 4D). Similarly, we observed enrichment of the rCap EC gene signature in gCap ECs found in human pulmonary fibrosis (Fig. 4E). We extended this analysis to spatial transcriptomics data of human health and IPF lung tissues, which revealed some enrichment of the rCap gene signature in areas within human IPF lungs (Fig. 4F), albeit not as abundant as that observed with rVC genes. We found that genes within the rCap EC population also showed enrichment in influenza injured gCap ECs (*56*) (Fig. 4G), as well as in gCap ECs following hyperoxia injury (*57*) (Fig. 4H), suggesting that the rCap gene expression program may relate to general capillary responses in broad injury settings.

To investigate potential paracrine signals induced by rCap ECs we performed CellChat analysis focusing on outgoing signals arising from these cells, comparing them to control gCap ECs (Fig. 4I, fig. S4C, and table S6). Distinct outgoing pathways from rCap ECs included COLLAGEN, TGFβ, ANGPT, and CSF signaling (with some similarity to the outgoing signals in the rVCs, suggesting common YAP/TAZ-driven programs) with rCap ECs signaling mostly to alveolar fibroblasts (AFs) (Fig. 4I). Notably, rCap ECs also showed reduced outgoing signals for several cell adhesion pathways such as CADM (cell adhesion molecules) and EPHB (EphrinB) (Fig. 4I), suggesting potential reduced adhesion responses, which might relate to the enhanced vascular permeability observed in the Aplnr-Lats1/2-cKO^tdtom^ lungs (fig. S1F).

### Lats1/2 deletion in Aplnr-expressing lung ECs is sufficient to promote profibrotic mesenchymal cell activation

**Fig. 5.**
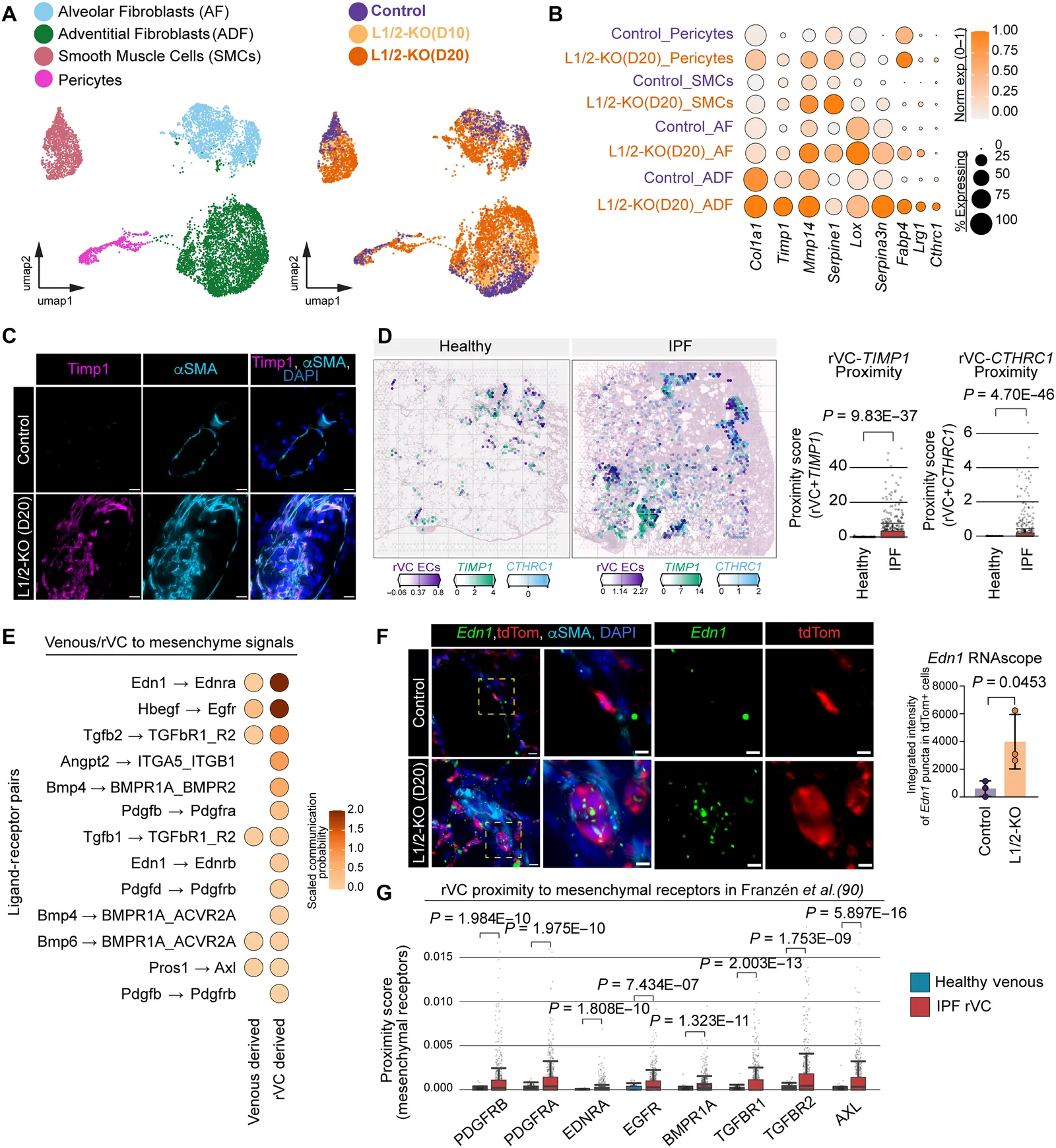
Lats1/2 inactivation in Aplnr-expressing cells drives distinct mesenchymal cell remodeling. (**A**) UMAP of all mesenchymal populations identified in our scRNA-seq experiment, split by condition and time point (*n* = 8228 cells). (**B**) Bubble plot of genes associated with fibrotic fibroblast activation split between control and Lats1/2-KO D20 mesenchymal cells. (**C**) IF for αSMA and Timp1 on control and Lats1/2-KO lungs (scale bars, 10 μm). (**D**) Spatial mapping of the Lats1/2-KO-rVC gene signature (purple) and TIMP1 expression (green) or CTHRC1 expression (blue) across a healthy and IPF sample from Franzén *et al.* (*90*). Quantification of the spatial proximity between the Lats1/2-KO-rVC gene signature and TIMP1 or CTHRC1 expression through a patch-based neighborhood analysis. (**E**) Bubble plot depicting the CellChat analysis of ligand-receptor pair comparison between Lats1/2-KO (D10 and D20 combined) rVCs and control venous ECs outgoing signaling to mesenchymal cells. Interaction probabilities were summed across targets, normalized and ranked, with color indicating scaled communication strength. (**F**) IF for αSMA, tdTom, and *Edn1* RNAscope on control and Lats1/2-KO lungs [scale bars, 10 μm and 5 μm (zoomed in)] with the bar plot showing the quantification of integrated puncta intensity (*n* = 3 per group). (**G**) Quantification of the spatial proximity between healthy venous ECs or IPF Lats1/2-KO-rVC signature positive ECs and mesenchymal receptors from Franzén *et al.* (*90*). For (F), values are summarized as means ± SEM and a two-tailed Student’s *t* test was performed. For (D) and (G), values are summarized as boxplots (interquartile range) with jittered single-cell points and a nonparametric Wilcoxon rank sum test was performed.

The marked αSMA+ cell remodeling we observed by histology within Aplnr-Lats1/2-cKO^tdtom^ lung lesions indicated an altered mesenchymal cell response that is initiated by EC-derived signals, a response further suggested by CellChat analysis of our scRNA-seq data. To better understand these responses, we subclustered the mesenchymal cells [8228 cells; annotated according to publicly available databases (*58*, *59*); fig. S5A and table S7], which revealed clusters of AF, adventitial fibroblasts (ADFs), SMCs, and pericytes. All mesenchymal populations exhibited progressive transcriptional shifts following Lats1/2 deletion, with responses that included extracellular matrix organization and matrix responses (Fig. 5A; fig. S5, B and C; and table S8). We observe the induction of fibroblast activation markers such as *Serpine1*, *Lrg1*, *Col1a1*, and *Mmp14* across most mesenchymal subpopulations (Fig. 5B and table S7). Consistent with the induced fibrotic response observed in the Aplnr-Lats1/2-cKO^tdtom^ lungs, ADFs harvested at day 20 express high levels of *Cthrc1*, a marker associated with collagen-producing fibroblasts in fibrosis (*60*), *Timp1*, which has been implicated in the development of bleomycin induced fibrosis in mice (*61*), and other fibrosis-related genes (Fig. 5B). Staining for Timp1 confirmed strong expression in mesenchymal cells surrounding tdTomato lesions in Aplnr-Lats1/2-cKO^tdtom^ lungs (Fig. 5C). We extended our analysis to explore the relationship between profibrotic fibroblasts and the vasculature in human healthy and IPF lung tissues. We found that the rVC gene signature score was enriched in areas that spatially associate with mesenchymal cells expressing *TIMP1* and *CTHCR1* and that these areas were elevated in IPF tissues (Fig. 5D).

To further explore the signaling relationship between rVCs and fibroblasts, we turned back to our ligand-receptor CellChat analysis of the scRNA-seq data from our mouse models (Fig. 3L), focusing on EC-derived factors predicted to signal directly to fibroblasts. A notable elevation of genes encoding profibrotic secreted factors was evident in rVCs, including Edn1, TGFβ, and PDGF, with these factors predicted to signal directly to fibroblasts (Fig. 5E). RNAscope staining for *Edn1* indicated elevated expression levels in tdTomato-expressing EC lesions in Aplnr-Lats1/2-cKO^tdtom^ lungs compared to controls (Fig. 5F). Analysis of human spatial RNA-seq data from human healthy and IPF lung tissues revealed that the rVC-associated gene score showed elevated spatial association in IPF tissues with mesenchymal cells encoding receptors predicted to receive profibrotic signals originating from the rVCs. These receptors include *TGFBR*, *PDGFR*, *EDNRA*, and others (Fig. 5G), suggesting direct paracrine communication between the vasculature and fibroblast in human IPF.

### Lats1/2 deletion in Aplnr-expressing lung ECs drives immune dysregulation, myeloid cell recruitment, and aberrant epithelial cell states

**Fig. 6.**
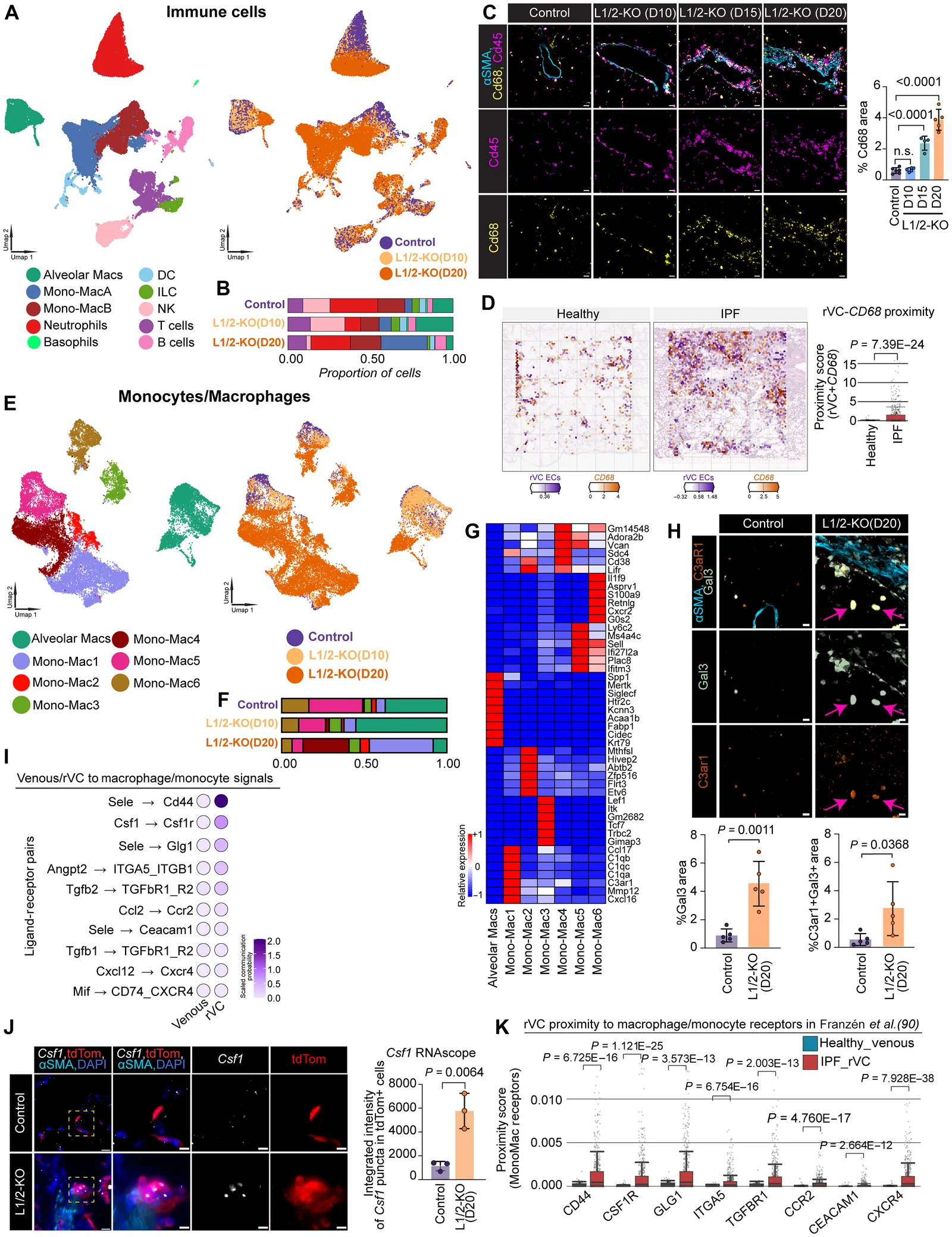
Distinct phagocytes are recruited to rVC lesions following Lats1/2 inactivation in Aplnr+ cells. (**A**) UMAP of immune cells from scRNA-seq (*n* = 53,954), shown by cluster (left) and condition/time point (right). (**B**) Proportion of immune cell populations. (**C**) IF for αSMA, Cd45, and Cd68 in control and Lats1/2-KO lungs (D10, D15, and D20; scale bars, 20 μm) with quantification of the Cd68+ area (*n* = 6 control; *n* = 4 D15 KO; *n* = 5 D10 and D20 KO). n.s., not significant. (**D**) Spatial mapping of the Lats1/2-KO rVC gene signature (purple) and CD68 (orange) in healthy and IPF lungs [Franzén *et al.* (*90*)], with patch-based neighborhood analysis. (**E**) UMAP of reclustered macrophages/monocytes (*n* = 24,107), shown overall (left) and by condition/time point (right). (**F**) Proportion of macrophage/monocyte subclusters across time points. (**G**) Heatmap of genes enriched in each macrophage/monocyte cluster. (**H**) IF for αSMA, Gal3, and C3ar1 in control and Lats1/2-KO D20 lungs (scale bars, 10 μm); arrows indicate Gal3/C3ar1+ cells, with quantification of the Gal3+ and Gal3/C3ar1+ area (*n* = 5 per group). (**I**) CellChat analysis of ligand-receptor interactions from Lats1/2-KO rVCs versus control venous ECs to macrophages/monocytes. Interaction probabilities were summed across targets, normalized and ranked, with color indicating scaled communication strength (**J**) IF for αSMA, tdTom, and *Csf1* RNAscope in control and Lats1/2-KO lungs (10 μm; inset, 5 μm) with quantification of the puncta intensity (*n* = 3 per group). (**K**) Spatial proximity analysis between venous ECs or rVC signature-positive ECs and immune receptors in spatial transcriptomics data from healthy and IPF lungs [Franzén *et al.* (*90*)]. Statistical analyses: For (C), (H), and (J), values are summarized as means ± SEM. For (D) and (K), values are summarized as boxplots (interquartile range) with jittered single-cell points. For (C), a one-way ANOVA was performed; for plots (D) and (K), a nonparametric Wilcoxon rank sum test was performed, and for (H) and (J), a two-tailed Student’s *t* test was performed.

Our histologic analysis of Aplnr-Lats1/2-cKO^tdtom^ lungs indicated that Lats1/2 deletion is sufficient to drive progressive immune cell recruitment to venous-associated lesions. To investigate these immune alterations, we subclustered all *Cd45*+ cells (53,954 cells) from our scRNA-seq data, which indicated distinct immune changes following Lats1/2 deletion (Fig. 6A). Notable differences included a progressive loss of natural killer (NK) cells, a major hallmark of IPF pathogenesis (*62*, *63*) and a progressive increase in myeloid cells (Fig. 6B). We found that 75% of leukocytes captured at the day 20 time point following Lats1/2 deletion fell under the myeloid family, which included macrophages, monocytes, and dendritic cells (DCs), with the most notable increase in monocytes and macrophage numbers following Lats1/2 deletion (fig. S6, A and B). We validated this by staining lung tissues for Cd68, a macrophage/monocyte marker, which showed a strong accumulation of Cd68+ cells within the venous-associated lesions in Aplnr-Lats1/2-cKO^tdtom^ lungs (Fig. 6C). We observed a similar relationship between venous ECs and macrophages in spatial RNA-seq data from human IPF, which showed a correlation between rVC gene signature scores and cells expressing the monocyte/macrophage marker *CD68* that was enhanced in IPF versus healthy lung tissues (Fig. 6D).

To better understand these cell responses, we subclustered monocytes/macrophages, which revealed six monocyte-macrophage subpopulations (*Ccr2/Csf1r/Cd14/Cd11b*^+^) (Mono-Mac1 to Mono-Mac6) and one Alveolar Macrophage cluster (*Marco/Mertk*^+^) (Fig. 6E) that showed different proportions depending on condition and time point (Fig. 6F), which we annotated on the basis of the expression of distinct and established markers (Fig. 6G, fig. S6C, and table S9). Most clusters were found to undergo a transcriptional shift in response to the Lats1/2 deletion; however, we observed that Mono-Macs1, Mono-Macs2, and Mono-Macs4 distinctly associated with the day 20 time point of Aplnr-Lats1/2-cKO^tdtom^ lungs (Fig. 6, E and F). These clusters exhibit induced expression of ligands (e.g., *Il1a*, *Il1b*, and *Il6*) and recruitment receptors (*Csf1r*, *Ccr5*, *Itgam*, and *Itgb1*) with established roles in regulating inflammatory responses (fig. S6D and table S9). GO enrichment revealed that these clusters are enriched for leukocyte migration, leukocyte proliferation, and myeloid leukocyte activation, signifying that they could be a combination of recruited and resident-expanded cells (fig. S6E). Alveolar macrophages showed a strong transcriptomic response following endothelial Lats1/2 inactivation, with several migratory and cytokine-response pathways enriched (fig. S6, F and G, and table S10). Differential gene expression analysis between all subclusters revealed that C3ar1, the receptor for Complement protein 3 (C3), was found to mark Mono-Macs1, Mono-Macs3, and Mono-Macs4 distinct to the day 20 Lats1/2 deleted lungs (fig. S6H and table S9). Immunohistochemical staining for C3ar1 together with Galectin-3, a macrophage/monocyte marker, revealed dual-positive cells localized within Lats1/2-KO (knockout) venous lesions (Fig. 6H). When examining human scRNA-seq data, we observed a similar induction of *C3AR1* expression in macrophage/monocytes in IPF (fig. S6I). Moreover, we observed a spatial association between cells enriched for the rVC gene score and cells expressing *C3AR1* in spatial transcriptomics data from healthy and IPF human lung tissues, with the proximity of these cells strongly elevated in IPF tissues (fig. S6J). *C3*, the gene encoding the C3ar1 ligand, was primarily expressed in ADFs and AFs with elevated levels observed in Lats1/2-KO lungs (fig. S5D), similar to the elevated levels observed in human IPF fibroblasts (fig. S5, E and F). CellChat analysis of the scRNA-seq data from our mouse models revealed an increase in the Lats1/2-KO lungs for fibroblast-derived C3 signaling, which was also observed in human IPF lungs (fig. S5, G and H). These observations suggest that the C3-C3ar1 signaling axis is a feature of the fibrotic niche and suggest an interdependent signaling network between ECs, fibroblasts, and macrophages.

Prior reports have suggested key roles for SPP1+ macrophages in fibrotic lung remodeling (*64*, *65*), prompting us to examine the expression of Spp1 in the macrophage/monocyte clusters, which showed increased expression in Alveolar Macrophage and Mono-Macs1 following Lats1/2 inactivation (fig. S6K). CellChat analysis revealed that a major receiver for the Spp1 ligand are the alveolar and ADFs in the Lats1/2-KO lungs (fig. S6L), which was also observed in human IPF samples (fig. S6M). In addition, we examined genes distinctly expressed in IPF-associated SPP1+ macrophages in macrophages accumulating with Lats1/2 inactivation. This analysis revealed that the pathogenic SPP1+ macrophage gene signature is enriched in several macrophage/monocyte populations that accumulate following Lats1/2 inactivation (fig. S6N), further supporting that endothelial Lats1/2 inactivation results in the recruitment and remodeling of pathogenic macrophages, which, in turn, can propagate fibrotic remodeling through signaling to the mesenchyme.

To gain insight into potential direct signaling communication between rVCs and monocytes/macrophages, we revisited our ligand-receptor CellChat analysis of the scRNA-seq data derived from our mouse models. Several notable secreted factors known to recruit, polarize, or modulate macrophages were found to be elevated in rVCs, including *Csf1*, *TGFβ*, and *Ccl2*, as well as adhesion molecules known to promote monocyte-endothelial tethering and rolling adhesion, such as *Sele* (Fig. 6I). RNAscope analysis of control and Aplnr-Lats1/2-cKO^tdtom^ lungs validated that the expression of *Csf1* was elevated in tdTomato-marked lesions developing following Lats1/2 inactivation (Fig. 6J). Analysis of spatial RNA-seq data from human healthy and IPF lungs similarly demonstrated a strong spatial association between ligand-receptor pairs (including CSF1-CSFR1) predicted to mediate rVC-monocyte/macrophage communication (Fig. 6K). Collectively, these data suggest direct EC signaling to innate immune cells that is elevated in the fibrotic niche.

**Fig. 7.**
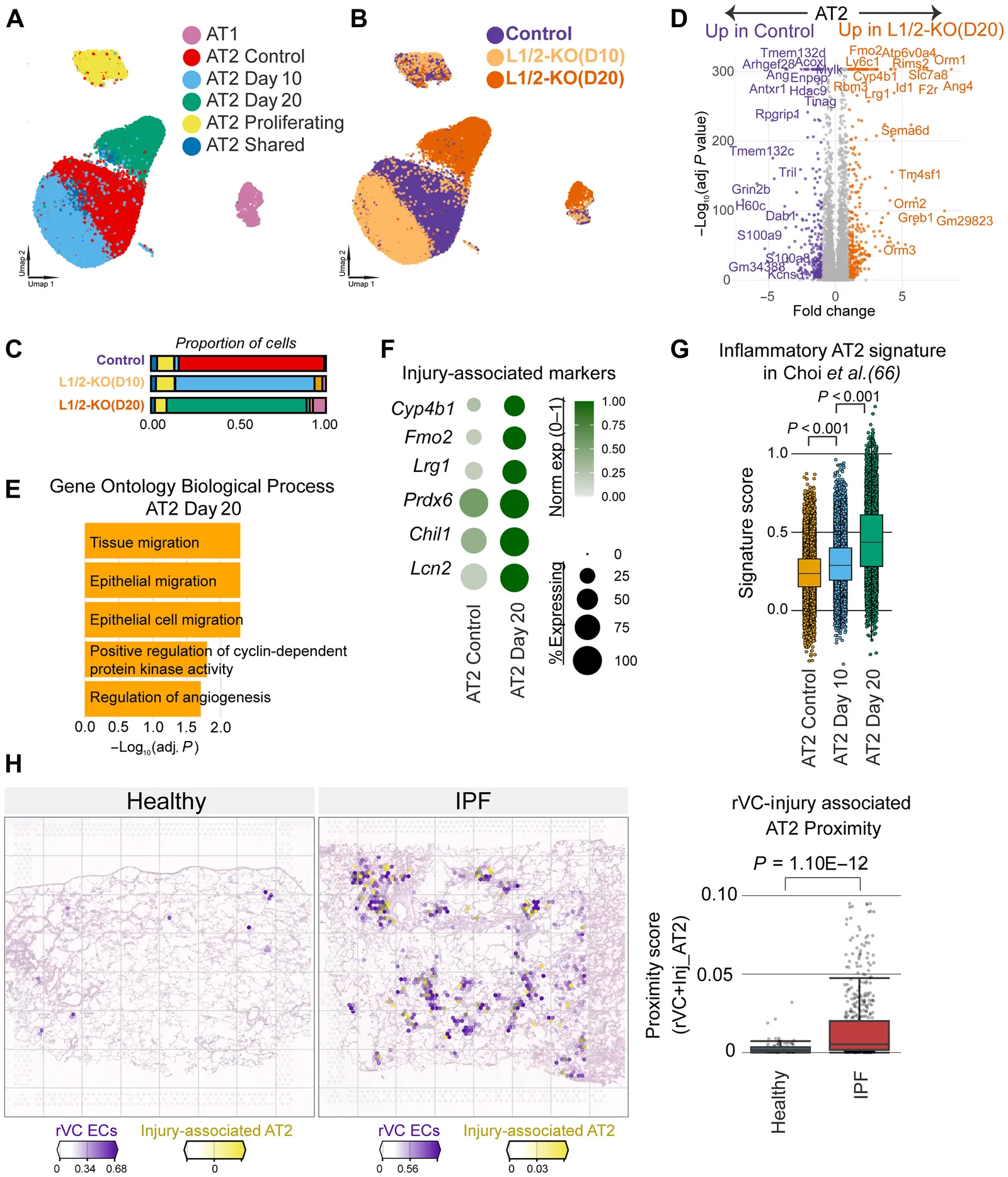
Lats1/2 inactivation in Aplnr-expressing cells drives distinct epithelial cell remodeling. (**A**) UMAP of alveolar epithelial cell clusters (*n* = 55,866 cells), along with (**B**) alveolar epithelial cells split per condition and time point. (**C**) Proportion analysis of all alveolar epithelial cells. (**D**) Volcano plot comparing genes differentially expressed across control and Lats1/2-KO D20 AT2 cells. (**E**) GO enrichment of genes distinctly expressed in AT2 cells identified in Lats1/2-KO D20 lungs. (**F**) Bubble plot depicting epithelial injury–associated markers across control and Lats1/2-KO D20 AT2 cells. (**G**) Signature enrichment for AT2 inflammatory signature [Choi *et al.* (*66*)] across control and Lats1/2-KO D10 and D20 AT2 cells. Data shown as box-and-whisker plots (interquartile range) with individual cells overlaid as jittered points. Differences were evaluated using one-way ANOVA, with *P* values adjusted using the Benjamini-Hochberg method. (**H**) Spatial mapping of the Lats1/2-KO-rVC gene signature (purple) and the AT2 Injury–associated gene signature (yellow) across a healthy and IPF sample from Franzén *et al.* (*90*) (left). Quantification of the spatial proximity between the Lats1/2-KO-rVC gene signature and the AT2 Injury–associated gene signature through a patch-based neighborhood analysis (right). Statistical significance was determined using a nonparametric Wilcoxon rank sum test.

Given the major remodeling of immune and mesenchymal cells within Aplnr-Lats1/2-cKO^tdtom^ lungs, we reasoned potential epithelial remodeling as well. To explore the impact on the epithelium, we reclustered epithelial cell populations captured in our scRNA-seq data (58,722 cells). Our analysis revealed that we captured both airway and alveolar epithelial cells, with the most prominent changes observed in the alveolar epithelium following Lats1/2 inactivation (fig. S7, A and B). Histologic analysis of Aplnr-Lats1/2-cKO^tdtom^ lungs did not reveal noticeable changes in AT1 or AT2 cell numbers (fig. S7C), suggesting transcriptomic shifts in these cell populations. Five distinct alveolar epithelial subclusters were observed in our scRNA-seq data with three unique to each time point (control and Lats1/2-KO D10 and D20), one proliferating, and one shared across all time points (Fig. 7, A to C).

Differentially expressed gene (DEG) analysis comparing control and Lats1/2-KO D20 AT2 cells revealed a distinct transcriptional response (Fig. 7D and table S11), including induction of *Cyp4b1* and *Fmo2*, enrichment of genes related to cell migration and actin cytoskeletal remodeling (Fig. 7E), and increased expression of genes encoding factors known to associate with epithelial injury, including *Lrg1*, *Prdx6*, *Chil1*, *Lcn2*, and *Il33* (Fig. 7F and table S12). Lats1/2-KO D20 AT2 cells were enriched for genes previously associated with a pro-inflammatory state that is acquired by AT2 cells following injury (*66*) (Fig. 7G) and have shared features with pre-alveolar type 1 transitional state (PATS) cells (*67*) (fig. S7D). A similar relationship between inflammatory AT2 cells and dysfunctional ECs was also observed in human lungs tissues as the AT2 inflammatory gene signature spatially associated with cells enriched for the rVC gene score in spatial transcriptomics data, with increased association observed in IPF compared to healthy lung tissues (Fig. 7H). These observations indicate that signaling responses initiated by EC dysfunction following Lats1/2 deletion can drive cellular remodeling that results in the induction of epithelial damage responses.

### Inhibition of YAP/TAZ-TEAD blocks the dysfunctional responses resulting from endothelial Lats1/2 inactivation

**Fig. 8.**
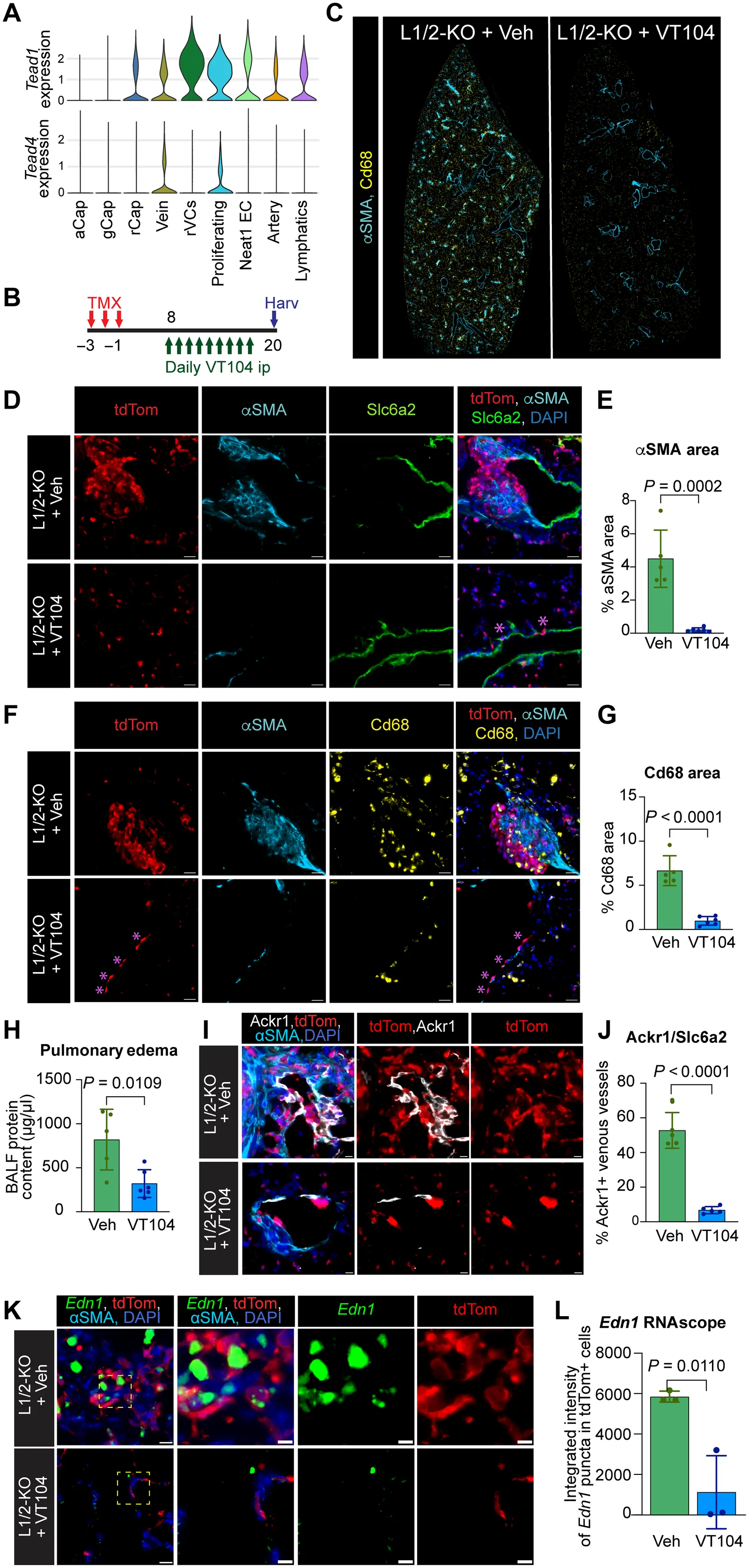
TEAD inhibition attenuates the development of lung lesions driven by Lats1/2 inactivation in Aplnr-expressing cells. (**A**) Violin plot of *Tead1* and *Tead4* expression across EC subclusters. (**B**) Schematic of the experimental setup for tamoxifen and VT104 delivery via intraperitoneal injection. ip, intraperitoneal. (**C**) IF for αSMA and Cd68 on Lats1/2-KO D20 lungs treated with vehicle or the TEAD inhibitor VT104 (scale bars, 500 μm). (**D**) IF for tdTom, αSMA, and Slc6a2 on vehicle-treated and VT104-treated Lats1/2-KO-D20 lungs (scale bars, 5 μm) (pink asterisk marks tdTom+ rVCs). (**E**) Quantification of the normalized area coverage for αSMA on vehicle-treated and VT104-treated Lats1/2-KO-D20 lungs, analyzed using a two-tailed Student’s *t* test (*n* = 5 vehicle-treated and 6 VT104-treated Lats1/2-KO lungs). (**F**) IF for αSMA, tdTom, and Cd68 on vehicle-treated and VT104-treated Lats1/2-KO-D20 lungs (scale bars, 20 μm) (pink asterisk marks tdTom+ rVCs). (**G**) Quantification of the normalized area coverage for Cd68 on vehicle-treated and VT104-treated Lats1/2-KO-D20 lungs (*n* = 5 vehicle-treated and 6 VT104-treated Lats1/2-KO lungs). (**H**) Protein quantification of BALF from vehicle-treated and VT104-treated Lats1/2-KO-D20 lungs (*n* = 5 vehicle-treated and 6 VT104-treated Lats1/2-KO lungs). (**I**) IF for αSMA, tdTom, and Ackr1 on vehicle-treated and VT104-treated Lats1/2-KO-D20 lungs (scale bars, 20 μm). (**J**) Quantification of Ackr1-positive vessels across the total number of Slc6a2-positive venous vessels (*n* = 5 per group). (**K**) IF for αSMA, DAPI, tdTom, and *Edn1* RNAscope on vehicle-treated and VT104-treated Lats1/2-KO-D20 lungs [scale bars, 10 μm and 5 μm (zoomed in)] (*n* = 3 per group). (**L**) Quantification of the integrated *Edn1* puncta intensity on vehicle-treated and VT104-treated Lats1/2-KO-D20 lungs (*n* = 3 per group). For (E), (G), (H), (J), and (L), values are summarized as means ± SEM and a two-tailed Student’s *t* test was performed.

We observed that the YAP/TAZ-associated transcription factors *Tead1* and *Tead4* were distinctly elevated in rCap ECs and rVCs that emerge following Lats1/2 inactivation (Fig. 8A) (*Tead2* and *Tead3* were not detected to be expressed), which combined with elevated nuclear YAP/TAZ levels (Fig. 1C), suggested a key role for YAP/TAZ-TEAD activity in the observed responses. This prompted us to test the effects of the small molecule inhibitor VT104, known to block YAP/TAZ-TEAD activity in vivo (*68*, *69*). For this, we treated Aplnr-Lats1/2-cKO^tdtom^ mice with tamoxifen, and 8 days later (a time point at which lesions start to become evident), we started dosing animals with either vehicle control or VT104. Inhibition of YAP/TAZ-TEAD showed a remarkable reduction in development of venous-associated lesions in Aplnr-Lats1/2-cKO^tdtom^ lungs (Fig. 8C), with a notable block in the accumulation of αSMA+ mesenchymal cells (Fig. 8, D and E) and macrophage recruitment (Fig. 8, F and G). VT104 treatment also attenuated the pulmonary edema resulting from Lats1/2 inactivation (Fig. 8H), suggesting a rescue of the alveolar-vascular barrier breakdown. VT104 treatment led to reduced levels of factors that mark expanding tdTomato lineage–traced rVCs within Aplnr-Lats1/2-cKO^tdtom^ lungs, indicating effectiveness in targeting activated ECs. This included reduced Ackr1 following VT104 treatment (Fig. 8, I and J), as well as reduced levels of factors predicted to mediate EC-derived paracrine signals, such as *Edn1* (Fig. 8, K and L) and *Csf1* (fig. S8, A and B). Thus, inhibition of YAP/TAZ-TEAD activity successfully attenuated the endothelial-derived defects resulting from Lats1/2 inactivation in Aplnr-expressing ECs, highlighting YAP/TAZ-TEAD as a therapeutic target in pulmonary EC dysfunction.

## DISCUSSION

**Fig. 9.**
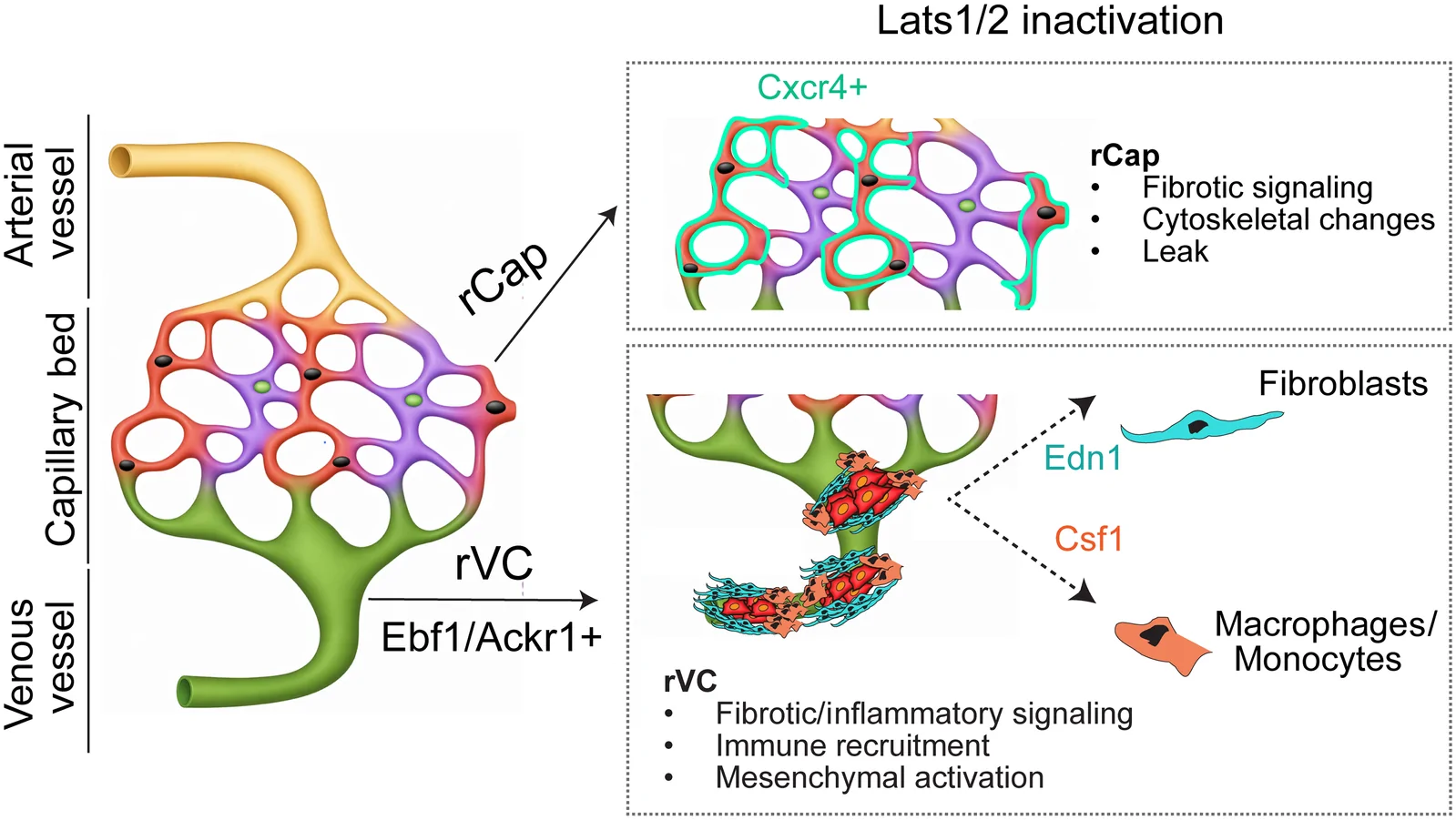
Schematic of rCap and rVC responses to Lats1/2 inactivation. Schematic representing the distinct responses emerging after Lats1/2 inactivation in Aplnr-expressing cells. Observations suggest that Capillary gCap ECs transition to a responsive capillary state (rCap) that exhibit cytoskeletal changes and fibrotic signaling activation. Observations suggest that a distinct population of responsive venous ECs (rVCs) signal to fibroblasts and monocytes/macrophages, directly contributing to the establishment of a fibrotic niche.

The pulmonary endothelium is increasingly recognized as a dynamic and disease-modifying component of the lung, integrating mechanical, inflammatory, and metabolic cues to shape local tissue responses. Although ECs have long been appreciated for their roles in vascular tone, barrier function, and leukocyte trafficking, recent single-cell and lineage tracing studies have revealed a far more complex role for the lung vasculature in injury responses and chronic lung diseases (*4*, *9*, *10*, *15*, *56*). Emerging evidence indicate that ECs undergo substantial remodeling during injury and fibrosis and has suggested that these states may contribute to maladaptive tissue repair (*10*–*15*). Prompted by these prior observations, we tested the consequences of hyperactivating the transcriptional regulators YAP/TAZ in lung ECs, which is a signaling axis predicted to associate with altered EC states following lung injury (*10*). Using a genetic model targeting Aplnr-expressing ECs, we demonstrate that deletion of the LATS1/2 kinases, which are major inhibitors of nuclear YAP/TAZ activity, results in distinct EC responses that are sufficient to drive progressive inflammation, stromal remodeling, and the establishment of a profibrotic microenvironment. Notably, divergent responses emerge for cells positioned within different vascular beds of the lung, with our data highlighting a venous-associated EC population that has fibrotic remodeling potential (Fig. 9).

A key finding from our study is the identification of distinct cellular trajectories resulting from Lats1/2 deletion using the Aplnr-CreERT2 model, which is a model that has been reported to primarily target gCap ECs within capillaries and postcapillary arterial and venous ECs in adult lungs of mice (*3*, *10*). This model thus offers more restricted targeting of lung ECs, compared to other endothelial Cre models, such as the Cdh5CreERT2 model, which have been used to study Hippo pathway signaling in other contexts (*30*, *46*). We found that the targeting achieved by the Aplnr-CreERT2 model marks a small percentage of ECs within large veins and arteries, consistent with previous reports examining Aplnr expression (*70*–*72*). These large vessel Aplnr lineage–labeled ECs showed heterogeneous profiles within the same vascular bed, suggesting distinct properties. Inactivation of Lats1/2 resulted in the unique expansion of these cells in veins. We carefully tracked this population and found a proliferative response coupled to EC migration, resulting in EC expansion within vein lumens or past the vessel basement membrane into the extravascular space. The expansion of these venous ECs was coupled closely to the recruitment of monocytes/macrophages, the local accumulation of αSMA+ and TIMP1+ mesenchymal cells, and fibrotic remodeling of the neighboring niche. The distinct expansion of these venous-associated cells raises many questions into their nature. Our analysis of markers associated with these cells, such as Ebf1, suggest that these cells exist at homeostasis and may have unique properties. Ebf1 expression has been reported in the lung, including in pulmonary vascular cells in embryonic development (*73*) and in disease-associated perivascular cells and fibroblasts (*13*, *74*, *75*), suggesting key roles for this factor in the Aplnr-expressing venous population. One hypothesis is that these cells may have progenitor properties as there are observations that Aplnr expression is associated with cardiac progenitors (*76*) and transient progenitor populations during the development of hematopoietic stem cells (*77*, *78*). Aligning with this idea, recent observations have suggested that pulmonary venous ECs have the capacity to proliferate and differentiate into capillary subpopulations (aCap and gCap ECs) in response to viral injury (*42*). However, additional experimentation would be needed to elucidate this idea.

ScRNA-seq demonstrated that the expanding venous population arising from Lats1/2 inactivation, which we termed rVCs, has a distinct transcriptional program. Genes selectively enriched in rVCs comprised those encoding sets of transcription factors, including *Ebf1* and *Meox2*. Ebf1 expressing rVCs emerging in Aplnr-Lats1/2-cKO^tdtom^ lungs were tightly associated with macrophage recruitment and fibrotic remodeling of the surrounding microenvironment, suggesting that these cells act as a local hub for initiating fibrotic responses. Several secreted factors were found to be expressed in rVCs with the potential for paracrine cellular remodeling, including fibrosis-associated growth factors, chemokines and immunomodulatory factors, and angiogenic factors. Moreover, we found the molecular program induced in rVCs resembled that of a recently identified venous-like population of ECs found to be expanded in human IPF lungs (*79*). This population, reported as a *COL15A1*-expressing venous population similarly expresses *EBF1* and is closely localized with fibrotic foci. Analysis of human lung spatial transcriptomics data suggest that rVCs are abundant in human IPF lungs and that these cells spatially associate with disease-associated monocytes/macrophages and profibrotic fibroblast. These observations thus raise the possibility that YAP/TAZ induced rVCs actively drive lung fibrotic remodeling. Consistent with this idea, several profibrotic secreted factors emanate from rVCs with predicted direct signaling to local stromal cells.

In addition to the venous response in Aplnr-Lats1/2-cKO^tdtom^ lungs, we observed a different response in gCap ECs located in the alveolar capillary bed, with the emergence of cells resembling capillary ECs that we termed rCap ECs. These data suggest that gCap ECs undergo a transcriptomic shift upon Lats1/2 inactivation, leading to the observed rCap EC response that includes the induction of injury-related factors, cytoskeletal changes that affect cell adhesion and cell junctions. Consistent with these responses, we observed notable vascular leak throughout Aplnr-Lats1/2-cKO^tdtom^ lungs, shown by enhanced Evans Blue leak and protein content in the BALF. We further found that Lats1/2 inactivation in human lung microvascular ECs drives a loss in adhesion and barrier functions in vitro, highlighting the repression of nuclear activity as an important event in the establishment and maintenance of the EC barrier, consistent with observations made during vascular development and function in other organs (*30*, *46*, *47*). When compared to publicly available scRNA-seq datasets from injured mouse or diseased human lungs, these cells closely resemble activated capillary gCap ECs found in hyperoxia-, bleomycin-, and influenza-treated lungs, suggesting that YAP/TAZ activation is linked to the injury response of gCap ECs. However, we acknowledge that our understanding of the origin or fate of rCap ECs remains incomplete from our work, and it is possible that there may be a relationship with the rVC population we identified. Additional studies would be needed to understand the hierarchy and relationship of these populations.

A prominent phenotype arising in the Aplnr-Lats1/2-cKO^tdtom^ lungs is the onset of fibrotic lesions, indicating that the lung endothelium (likely the rVC subpopulation that expands) is sufficient to drive fibrotic remodeling. We observed the recruitment of αSMA+ mesenchymal cells and the local activation of fibroblasts, with our scRNA-seq analysis suggesting that this response may involve multiple mesenchymal populations. Immune cell remodeling was also distinctly observed in these fibrotic lesions, with local macrophages being observed early in lesion development, suggesting a cellular network that is coupled to immune recruitment. Several immune regulating cytokines and chemokines (e.g., *Csf1*), along with immune recognizing cell surface proteins (e.g., *Sele*) were found to be elevated with Lats1/2 inactivation, suggesting direct immune remodeling by ECs. It is worth noting that Lats1/2 have described roles in maintaining nuclear integrity (*80*) and loss of Lats1/2 activity has been recently linked to Lamin A dysregulation and the onset of a senescence-associated state (*47*), which could be linked to the observed inflammatory responses. Also interesting is that the macrophage populations accumulating near rVC lesions following Lats1/2 inactivation express C3ar1, the receptor for complement C3. The C3-C3ar1 axis is known to play key roles in directing lung inflammatory responses, including trained immunity in the lung that results in an innate immune memory (*81*). Such immune memory is documented to be protective in some settings (e.g., pathogen infections) but could potentially contribute to aberrant inflammatory responses associated with chronic injury that drives fibrotic remodeling. Consistent with this idea, antagonism of C3ar1 has been shown to reduce fibrosis following bleomycin injury of mouse lungs (*82*). Further relevance is suggested by our observed similarities in the positioning of C3ar1 cells near rVC-enriched areas in spatial transcriptomics data from human IPF, raising the possibility that inflammatory responses may be orchestrated in part as a result of defective vascular phenotypes.

Our model was designed to test the consequences of persistently activated nuclear YAP/TAZ, which revealed unexpected divergence in phenotypes across capillary and venous compartments of the lung vasculature. YAP/TAZ activity is tightly linked to changes in tissue mechanics, and thus we can speculate that changes in mechanical forces may associate with the cellular and molecular changes we have observed. Thus, changes in lung vascular mechanics resulting from disrupted tissue architecture may be coupled to the expansion of rVCs or the persistent activation of rCap ECs in disease. Such changes may occur following chronic injury conditions or even with the normal aging process. Lung diseases, such as IPF, are linked to progressive mechanical defects that associate with these characteristics and are reported to be linked to hyperactive YAP/TAZ activity (*25*). Our observations suggest that there may be temporal consequences to the aberrant activation of YAP/TAZ in the vasculature that results in venous expansion, local inflammation, and fibroblast remodeling that reinforce signaling axes that promote the aberrant spread and movement of rVCs. Thus, targeting aberrant YAP/TAZ activity in such fibrotic remodeling presents a potential therapeutic strategy. YAP/TAZ are known to associate with several DNA binding factors, most notably the TEAD family of transcription factors (*24*). Accordingly, we demonstrate that the pharmacological inhibition of YAP/TAZ-TEAD signaling using the TEAD inhibitor VT104 prevented all major phenotypes observed in the Aplnr-Lats1/2-cKO^tdtom^ lungs, including blocking the accumulation of macrophages and fibroblasts and the development of fibrotic lesions. However, the effects on established fibrotic foci remains to be tested. We further note that we used a systemic delivery approach for TEAD inhibition in our experiments and thus given the reported roles for YAP/TAZ in lung epithelial regeneration and fibroblast responses (*22*, *25*, *83*), TEAD inhibition may also affect phenotypes more broadly. Nevertheless, our observations indicate that VT104 targets the aberrant gene expression in ECs arising from Lats1/2 inactivation, indicating potency in ECs.

Collectively, our study reveals that precise regulation of the LATS1/2-YAP/TAZ axis is essential for maintaining pulmonary endothelial homeostasis. Loss of this regulation precipitates venous and capillary endothelial transitions that mirror those observed in multiple lung disorders and demonstrate that endothelial dysfunction is sufficient to drive stromal activation, immune recruitment, vascular remodeling, and epithelial injury. These findings reshape our understanding of endothelial plasticity in lung disease and position endothelial YAP/TAZ activation as a central mediator of pulmonary EC function. Moreover, this work highlights opportunities for early therapeutic intervention aimed at restoring lung vascular homeostasis and preventing cellular cascades that drive lung chronic dysfunction.

## MATERIALS AND METHODS

### Mice

All animal experiments were performed according to protocols approved by the Institutional Animal Care and Use Committee (IACUC; protocols PROTO201800236, PROTO201800389, and PROTO201900054) at Boston University and conforming to the Animal Research: Reporting of In Vivo Experiments (ARRIVE) guidelines. Mice used in the study were generating by breeding Aplnr-CreERT [Tg(Aplnr-Cre/ERT2)] (*37*) (kindly provided by K. Red-Horse, Stanford University) with Rosa26-flox-stop-flox-tdTomato (Jax strain 007914) mice (*40*). These mice were then crossed to flox-Lats1-flox (Jax strain 024941) and flox-Lats2-flox (Jax strain 025428) mice (*38*, *39*). All mice used in these experiments were 8 to 16 weeks of age, had access to food and water ad libitum and were on a 12-hour/12-hour light/ dark cycle, an ambient temperature of 25 to 25.6°C, and a humidity of 46 to 49%. Tamoxifen (Sigma-Aldrich, T5648-5g) was prepared in corn oil (Sigma-Aldrich, C8267-2.5L) and administered at 75 mg of tamoxifen/kg body weight, daily for 3 consecutive days intraperitoneally. Tissues were analyzed at 10, 15, or 20 days following the last dose of tamoxifen. VT104 (Sigma-Aldrich, SML3445) was dissolved in dimethyl sulfoxide (Fisher, D136-1) and then combined with PEG-400 (polyethylene glycol, molecular weight 400; Sigma-Aldrich, PX1286B-2), 1x phosphate-buffered saline (PBS; Corning 21040CV), and Tween 20 (Sigma-Aldrich, P1379) (ratio: 40%-55%-5%) and given at 10 mg/kg daily by intraperitoneal injection. BALF was isolated posteuthanasia by ligating a blunt end needle in the trachea and washing the lung once with 1 ml of ice-cold PBS, and protein measurements were performed with a BCA kit (Fisher, 23227). Hydroxyproline measurements were performed 20 days after the last dose of tamoxifen, by perfusing, harvesting, and homogenizing the left lobe using a TissueLyser 2 (Qiagen), and a commercially available kit was used to measure hydroxyproline content (Sigma-Aldrich, MAK569). Hydroxyproline content data are expressed as micrograms of collagen per milligram of left lobe homogenate (μg/mg).

### Human lung tissue analyses

Lung tissues were obtained with the help of S. Huang (University of Michigan). IPF lung tissues were collected from explanted lungs from IPF patients at the time of lung transplantation following written informed consent. The study was approved by the University of Michigan Institutional Review Board (Ann Arbor, MI, USA; HUM00105694), and IPF diagnoses were established using clinical and pathological criteria and confirmed by multidisciplinary review. Normal control lungs were obtained from deceased donors whose lungs were not suitable for transplantation, with family consent for research use (Gift of Life, Michigan). No financial compensation was provided to donors or their families. Tissues were fixed in 4% paraformaldehyde and then paraffin embedded. In this study, we used lung tissues from five healthy donors (two females and three males) and six IPF (three females and three males). All donors were between the ages of 42 and 73 years old.

### Evans Blue Permeability Assays

Evans Blue was performed as previously described (*84*). Mice were anesthetized with a ketamine-xylazine solution (100 and 10 mg/kg, respectively), and then 100 μl of 1% Evans Blue solution (Fluka, 46160) in sterile PBS was injected retro-orbitally. Mice were euthanized after 30 min, and the lung was harvested, minced, and incubated in formamide for 48 hours at 68°C, after which the homogenate was analyzed under a Molecular Devices SpectraMax iD3 spectrometer.

### Cell culture

Human lung microvascular endothelial cell (HLMECs) lines were purchased from Cell Applications (540-05a) or ANGIO-PROTEOMIE (cAP-0032) and maintained in an EC growth basal medium supplemented with a microvascular EC growth kit (Sigma-Aldrich, C22010). Cells were grown on the inserts until they became confluent then treated with LATS1/2 or scramble siRNA (Horizon Discovery/Dharmacon, L-004632-00-0020 and L-003865-00-0020) for 48 hours. Fluorescently labeled dextran (Fisher, FD4-100 mg) was added at the apical compartment, and after an hour, samples from both the apical and basal compartments were analyzed on a spectrometer. TEER measurements were performed in triplicate for each well (Sigma-Aldrich, MERS00002). All the experiments were performed with cells within three to five passages.

### Immunohistochemistry

Formalin-fixed paraffin-embedded (FFPE) mouse lungs were cut in serial sections (5 μm). Sections were deparaffinized using a standardized protocol and blocked with 10% donkey serum (Sigma-Aldrich, S30-100ML) combined with rodent antibody blocking solution (Biocare Medical, RBM961L). Staining was performed using primary antibodies diluted in 5% donkey serum and incubated at 4°C overnight, and then washed and incubated with secondary antibodies and 4′,6-diamidino-2-phenylindole (DAPI) for an hour. Antibodies used are listed in table S13. For other stains, the following reagents were used: Shandon Harris Hematoxylin (Fisher, 6765003), Richard-Allan Scientific Eosin-Y with Phloxine (Fisher, 22050198), Picrosirius Red Stain Kit (Fisher, NC9908782), and trichrome stain (Masson) (Sigma-Aldrich, HT15-1KT). For RNAscope staining, slides were deparaffinized using a standardized protocol, processed using H_2_O_2_ and Protease Plus (ACD, 322330), stained for Edn1-mRNA (ACD, 435221) and Csf1-mRNA (ACD, 315621), and visualized using RED detection reagents (ACD, 322360). Then, slides were blocked with 10% donkey serum and incubated with the primary antibodies at 4°C overnight and then washed and incubated with secondary antibodies and DAPI for an hour. Slides were dehydrated and mounted using FluorSave (Sigma-Aldrich, 345789-20ML). Actin staining on fixed cells was performed using Alexa Fluor 488–Phalloidin (Cytoskeleton, SKU:PHDG1). Images were captured using a Zeiss Axio Scan.Z1 microscope with the Zeiss ZEN 3.1 Blue software or with a Zeiss Axio D1 observer inverted microscope with the Zeiss ZEN 3.1 Blue software. Image analysis was performed with CellProfiler (4.2.8) and EBImage (4.46.0).

### EdU labeling

EdU (Biosynth, NE08701) was prepared at 5 mg/ml in sterile PBS (Fisher, 21040CV), and 100 μl was given intraperitoneally for 3 consecutive days before harvesting. Lungs were harvested, formalin fixed, paraffin embedded, sectioned, and processed as above, and EdU detection was performed (Fisher, C10632).

### Single-cell RNA sequencing data generation

Lungs from 8- to 14-week-old male and female (four control, three Aplnr-Lats1/2-cKO^tdtom^ day 10, and two Aplnr-Lats1/2-cKO^tdtom^ day 20) mice were collected and processed into single-cell suspensions following established procedures. In brief, mice were euthanized and perfused with 15 ml of cold PBS through the right ventricle, and the lungs were inflated with Dulbecco’s modified Eagle’s medium (DMEM) containing collagenase (0.2 mg/ml) and DNase I (100 U/ml). Tissues were incubated for 2 min at room temperature, excised, finely minced, and then digested in an additional 30 ml of enzyme solution at 37°C for 35 min on a rotating platform. Digestion was halted by adding DMEM with 10% fetal bovine serum, and the suspension was filtered through a 40-μm strainer to remove debris. Red blood cells were lysed by resuspending the pellet in 1.5 ml of lysis buffer for 90 s, followed by dilution in 9 ml of PBS. The homogenate was then incubated with anti-Cd45 (Miltenyi, 130110618), incubated for 30 min at 4°C, and then passed through an isolation column (Miltenyi, 130042401). Cells were then washed and resuspended in 0.2 ml of 1× PBS containing 0.04% bovine serum albumin before submission to the Single-Cell Sequencing Core. Cell counts and viability were determined using a manual hemocytometer.

Single-cell suspensions of dissociated mouse lung were loaded on 10x Genomics GEM-X 3′ Chip at concentrations varying between 320,000 and 1,380,000 cells/ml with a viability between 70 and 88% for a targeted cell recovery of 10,000 cells per sample and processed using the Chromium GEM-X Single Cell 3′ Kit v4 and Dual Index Kit TT according to the manufacturer’s protocol (10x Genomics, CA, USA). The 10x Genomics Chromium X controller partitioned cells into gel bead-in-emulsions (GEMs) that were transferred to strip tubes and subsequently underwent reverse transcription. The samples were then stored in a −20°C freezer overnight. Samples were thawed at room temperature before addition of recovery reagent to break the GEMs, followed by amplification (11 cycles) and purification of double-stranded cDNA. Purified cDNA underwent fragmentation, end repair, A-tailing, and adaptor ligation before polymerase chain reaction amplification (10 to 14 cycles) to produce dual-indexed libraries with Illumina P5 and P7 sequences. The size distribution and molarity of amplified libraries were assessed using the Bioanalyzer High Sensitivity DNA Assay (Agilent Technologies, Lexington, MA, USA). The libraries were then pooled and sequenced on an Illumina NextSeq 2000 instrument at a 900 pM input with 2% PhiX spike in using a P4 100 cycle flow cell (Illumina, San Diego, CA, USA) resulting in ∼4000 to 63,000 mean reads per cell.

10x Genomics scRNA-seq data generated in our study is available in the National Center for Biotechnology Information (NCBI) Gene Expression Omnibus (GEO) database under identifier GSE316804.

### Single-cell RNA preparation

Reads were demultiplexed, aligned to a customized mm10 (version 2020-A) reference genome, and quantified using the GENCODE vM23/Ensembl 98 gene annotation via CellRanger (v6.1.2) with the --include-introns flag. The customized reference genome was created by augmenting the mm10 with the sequences of EYFP (enhanced yellow fluorescent protein) and tdTomato using cellranger mkref. The output matrices were imported into the SCTK-QC quality control pipeline from the SingleCellTK package (version 2.12.2) to generate a comprehensive set of quality control metrics including the library size, mitochondrial gene expression, and doublet prediction (*85*). Cells were excluded on the basis of high mitochondrial gene expression (>6% across all cells), low unique molecular identifier (UMI) counts (below the fifth percentile across all cells), low detected gene counts (below the fifth percentile across all cells), high decontX contamination score (>0.25), low library complexity (cells in which the top 200 most highly expressed genes accounted for more than 75% of the total UMI counts), and positive doublet predictions identified by more than one independent doublet-detection algorithm (*86*). After exclusion of low-quality cells, 135,193 high-quality cells across 15 samples were used in all downstream analyses.

The dataset was analyzed using the Seurat workflow v5.0.3 (*87*). To account for variability in sequencing depth, decontX counts were normalized using the LogNormalize method with a scale factor of 10,000 via the NormalizeData() function and scaled using ScaleData(). Highly variable features were identified using the variance-stabilizing transformation (vst) method implemented in FindVariableFeatures(), selecting the top 5000 highly variable genes for dimensionality reduction and clustering. Linear dimensionality reduction was performed using principal components analysis (PCA) on the scaled expression values of the highly variable genes via the RunPCA() function. The number of principal components (PCs) retained for downstream analyses was determined by inspection of the elbow plot and by selecting PCs capturing ∼95% of total variance. Seurat clusters were annotated by predicting cell types using LungMAP cellRef v0.1.0 (celltype_level3) as a reference (*88*). On the basis of predicted identities, the dataset was partitioned into four major subpopulations: Epithelial, Endothelial, Mesenchymal, and Immune. Each subpopulation was reprocessed independently by identifying the top 5000 highly variable genes, performing the PCA on the scaled expression values, and selecting PCs that together explained ∼90% of total variance. These PCs were again used for graph-based clustering with FindNeighbors() and FindClusters() (resolution = 0.1) and subsequent visualization with RunUMAP(). EC clusters with a tdTomato UMI count of 1 and above were classified as positive.

### Transcriptomics analysis

All packages used for scRNA-seq analysis can be found in table S14.

#### General plots

Heatmaps were generated by calculating the average gene expression for the selected gene across each cell type subcluster set using Seurat’s AverageExpression function on log-normalized data. Then, expression values were log-normalized on a per-gene basis using min-max scaling and linearly transformed to a range of −1 (lowest) to +1 (highest). Violin plots were generated with Seurat’s VlnPlot function by displaying expression distribution. Bubble plots were generated using Seurat’s DotPlot function by extracting each gene’s per-group average expression and percentage of cells expression. For visualization, average expression was log normalized on a 0 (lowest) and 1 (highest) scale per gene, whereas dot size represents the percent of cells expressing the gene. UMAPs depicting gene expression were generated using Seurat’s FeaturePlot, by overlaying each gene’s normalized RNA expression onto the two-dimensional UMAP.

#### Per-cluster pathway GO enrichment

GO enrichment was performed by first identifying DEGs in the cluster of interest using Seurat’s FindMarkers, after removing ribosomal and mitochondrial genes. Significant genes (adjusted *P* < 0.05) were mapped to Entrez IDs and subjected to GO analysis via clusterProfiler. Enriched GO terms were ranked by adjusted *P* value and plotted.

#### Signature enrichment

Signature enrichment was performed by first generating a gene list (see below), which was processed against the genes present in the dataset by computing a per-cell module score using Seurat’s AddModuleScore. Module scores were then extracted and plotted as boxplots with overlaid jittered single-cell points. Statistical significance was assigned by using the global Kruskal-Wallis test, followed by pairwise Wilcoxon rank sum tests.

#### Dataset preparation

For the Adams *et al.* (*48*), Raslan *et al.* (*10*), Hurskainen *et al.* (*57*), Niethamer *et al.* (*56*), and Habermann *et al.* (*50*) datasets, all ECs were subclustered using canonical endothelial markers (*CDH5/Cdh5* and *PECAM1/Pecam1*) and then further annotated for each endothelial subpopulation using described endothelial markers (*1*) and markers from LungMAPS (*89*). Following EC subtype annotation, arterial and venous ECs were merged into a Large Vessel category, whereas aCap and lymphatics ECs were removed, with the remaining cells split according to disease type.

#### Gene signatures

The gene signatures from the Raslan *et al.* (*10*) and Adams *et al.* (*48*) datasets, were generated by first identifying cells of interest based on their expression of Ackr1 (for Raslan *et al.*) and COL15A1 (for Adams *et al.*), followed by differential gene expression (genes expressed by at least 50% of the population with a 0.75 log fold change between groups) comparing all ECs to generate lists of up-regulated genes for these cells. The rVC and rCap signatures were generated by performing DEG analysis within the endothelial subcluster and then selecting up-regulated DEGs for either cluster. For the inflammatory AT2 signature, the unmodified gene list was taken from Choi *et al.* (*66*). The IPF SPP1 pathogenic macrophage signature for Morse *et al.* (*64*) was generated by first identifying cells of interest based on their expression of *SPP1* followed by differential gene expression within all macrophages to generate lists of up-regulated genes for these cells. The PATS signature was generated from the unmodified gene list for PATS is taken from Kobayashi *et al.* (*67*). All gene signatures can be found in table S3.

#### CellChat

CellChat (1.6.1) was performed by first splitting the dataset into control and KO (day 10 and day 20 merged). To reduce instability from small clusters, groups with fewer than 13 cells were removed before analysis. Communication probability was inferred using the CellChat standard pipeline and summarized at the pathway level and aggregated into condition-specific communication networks. For generating the circle plots, communication weight matrices that contain aggregated interaction strengths between sender and receivers were extracted. Circle plots were generated using the CellChat netVisual_circle, with the edge thickness scaled by sender-receiver interaction weight (communication strength). For the heatmap comparing outgoing interactions, the total outgoing weight from each sender-receiver pair was computed and then compared to their representative control (rCap versus gCap and rVC versus Venous). Differences were visualized as heatmaps where each tile corresponds to a receiver, and the fill color represents signaling weight difference. For the pathway comparison bubble plot, pathway-level communication probabilities for each sender (to all receivers) were computed and then compared to their representative control (rCap versus gCap and rVC versus Venous). For statistical significance, a two-sided Wilcoxon rank sum test was used, and *P* values were corrected for multiple testing using the Benjamini-Hochberg method [pathways with false discovery rate (FDR) *q* < 0.05 were considered significant]. Results are visualized as a bubble plot with bubble size reflecting the magnitude of pathway change between groups. For the receiver-specific comparison bubble plot, the pathway-level communication probability for each sender x receiver x pathway combination was computed and plotted as a dot plot with bubble size indicating the probability magnitude and color signifying the sender.

### Preprocessing of spatial Visium RNA-sequencing data

Data were downloaded from https://ebi.ac.uk/biostudies/studies/S-BSST1410 and described by Franzén *et al.* (*90*), with the article and data licensed under a Creative Commons Attribution 4.0 International License (http://creativecommons.org/licenses/by/4.0/). The cell2location annotation was obtained from “hs_visium_stutility_obj.rds.” SCANPY (version 1.11.5) and Squidpy (1.6.5) are used for preprocessing. The Space Ranger output files and the corresponding histology images were merged into a single anndata object. Quality control parameters were assessed, and the data were filtered to remove spots with less than 800 counts, more than 45,000 counts, less than 800 genes, or fewer than 25 cells. To filter Visium spatial data, we implemented a quality control pipeline using Squidpy to segment and count cellular units within each capture spot. High-resolution hematoxylin and eosin (H&E) images were first preprocessed with a Gaussian smooth (sigma = 1) and segmented via a Watershed algorithm to define individual cellular boundaries. By scaling the transcriptomic coordinates to the image pixel space, we extracted the number of unique segmentation labels underlying each spot. Last, we applied a strict filtering threshold, retaining only those spots containing more than 30 segments to ensure sufficient cellular density for reliable downstream analysis and deconvolution.

### Spatial proximity analysis

To quantify the spatial association between specific cell populations and fibrotic niches, we calculated a spatial proximity score *P* for each Visium tissue patch. This metric accounts for the physical adjacency of spots on the hexagonal grid rather than simple global correlation.

#### Graph construction

For each tissue patch, a spatial adjacency matrix *W* was constructed using the squidpy library. Spots were defined as neighbors $W_{\mathrm{ij}}=1$ if they shared a direct border on the Visium grid; otherwise, $W_{\mathrm{ij}}=0$.

#### Score calculation

The proximity score between two variables, *A* (e.g., *rVC* gene score abundance) and *B* (e.g., cell gene marker abundance), was defined as

$$P\left(A,B\right)=\frac{v_{A}^{T}{\mathrm{Wv}}_{B}}{\sum W}$$

where $v_{A}$ and $v_{B}$ are vectors representing the normalized expression or cell abundance scores across all n spots in the patch. The numerator represents the total interaction intensity between adjacent spots. The denominator is the total number of edges in the spatial graph, serving as a normalization factor for patch size and connectivity.

Statistical significance was determined using a nonparametric Wilcoxon rank sum test (Mann-Whitney *U* test).

#### General plots

To visualize the spatial distribution and colocalization of cells, packages cell2location and scanpy were used, by first extracting the raw expression values for each gene/signature for each sample. Then, spatial plots were generated by overlaying the normalized gene/signature expression onto high-resolution H&E tissue images. For analysis between healthy and IPF cell-cell proximity, CPE/CDH5-positive cells were first annotated as venous, whereas the rVC gene signature was used to identify rVCs. The spatial proximity analysis (above) was then performed to generate the proximity scores, which were then plotted as bar plots. All packages used for spatial sequencing analysis can be found in table S14.

### Public transcriptomics datasets

Publicly available datasets used in this study can be found as follows: Adams *et al.* (*48*) (GSE136831), Raslan *et al.* (*10*) (GSE264162), Morse *et al.* (*64*) (GSE128033), Habermann *et al.* (*50*) (GSE1135893), Hurskainen *et al.* (*57*) (GSE151974), Niethamer *et al.* (*56*) (GSE262927), and Franzén *et al.* (*90*) (S-BSST1410).
