# Supplementary material for: LATS1/2 inactivation drives a distinct venous endothelial cell response that contributes to fibrotic remodeling of the lung
Source: Sci Adv. 2026 Aug 28;12(35):eaef8468. doi: 10.1126/sciadv.aef8468 (PMC13524055; doi:10.1126/sciadv.aef8468)
Supplement: Supplementary file 1 [file sciadv.aef8468_sm.pdf]

Science Advances
AAAS

Supplementary Materials for

LATS1/2 inactivation drives a distinct venous endothelial cell response that contributes to fibrotic remodeling of the lung

Konstantinos Kontodimas *et al.*

Corresponding author: Giovanni Ligresti, ligresti@bu.edu; Xaralabos Varelas, xvarelas@bu.edu

Sci. Adv. **12**, eaef8468 (2026)
DOI: 10.1126/sciadv.aef8468

The PDF file includes:

Figs. S1 to S8
Legends for tables S1 to S14

Other Supplementary Material for this manuscript includes the following:

Tables S1 to S14

Fig S1

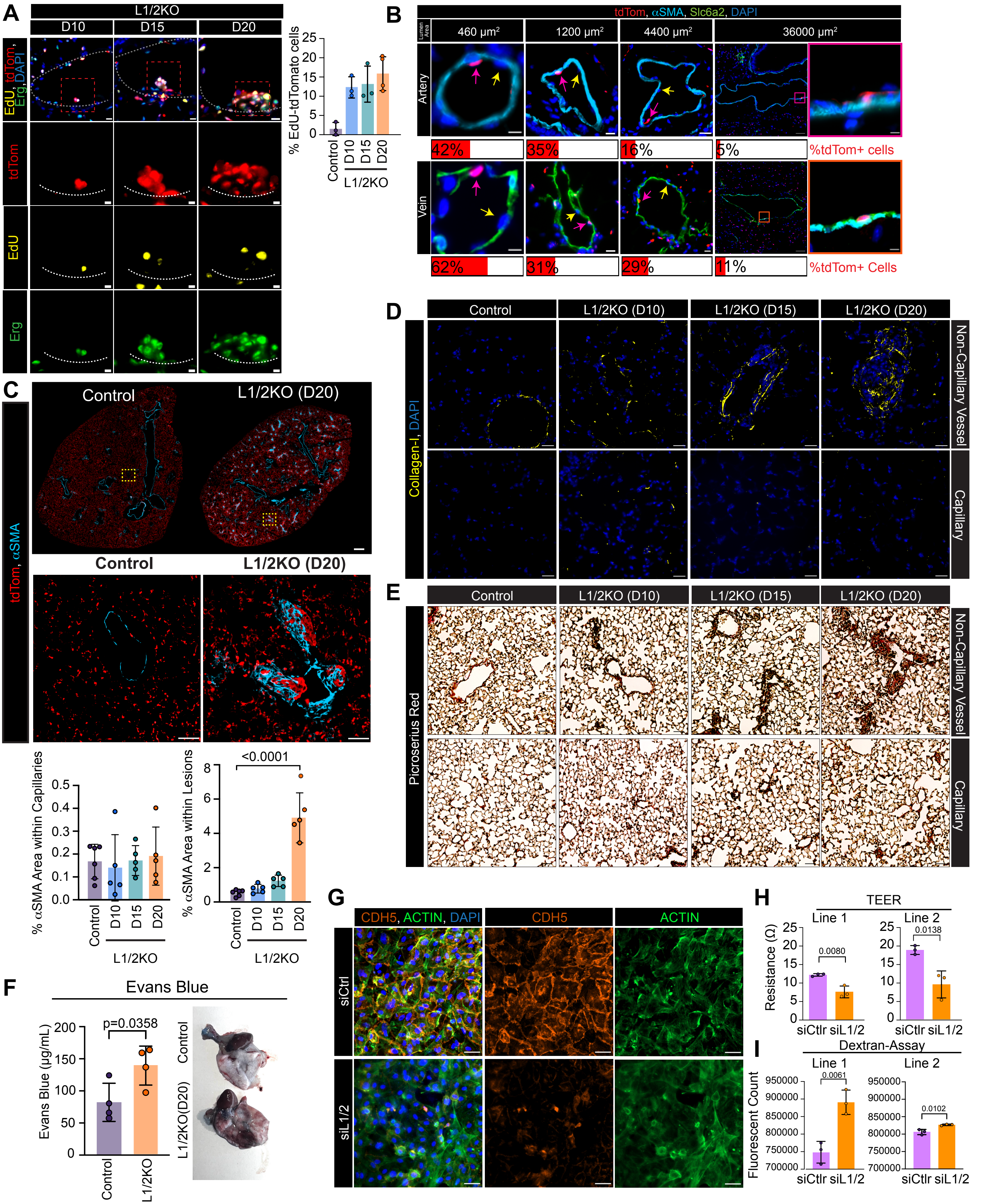
A
L1/2KO
D10
D15
D20
EdU, tdTom, Erg, DAPI
tdTom
EdU
Erg
% EdU-tdTomato cells
Control
L1/2KO
B
tdTom, αSMA, Slc6a2, DAPI
Lumen Area
460 μm²
1200 μm²
4400 μm²
36000 μm²
Artery
Vein
42%
35%
16%
5%
%tdTom+ cells
62%
31%
29%
11%
%tdTom+ Cells
C
Control
L1/2KO (D20)
tdTom, αSMA
% αSMA Area within Capillaries
% αSMA Area within Lesions
<0.0001
D
L1/2KO (D10)
L1/2KO (D15)
Collagen-I, DAPI
Non-Capillary Vessel
Capillary
E
Picroserius Red
F
Evans Blue
p=0.0358
Evans Blue (μg/mL)
L1/2KO(D20)
G
CDH5, ACTIN, DAPI
CDH5
ACTIN
siCtrl
siL1/2
H
TEER
Line 1
Line 2
Resistance (Ω)
0.0080
0.0138
siCtlr
I
Dextran-Assay
Fluorescent Count
0.0061
0.0102

***Fig S1*. (A)** IF staining for tdTom, ERG and EdU on paraffin-embedded tissues from Control and L1/2-KO mice at D10, D15 and D20 post tamoxifen treatment (large scale bar 10µm, zoom-in scale bar 5µm) and quantification of EdU-tdTom positive cells a (n=3 Control, D10 and D15 and n=4 Day20). **(B)** IF for Slc6a2, tdTom and αSMA in control lungs with the area of the respective vessel lumen listed above the images. Purple arrows mark tdTom labelled cells and yellow arrows mark non-labeled cells within the same vessel (scale bars in order of columns: 5µm, 20µm, 20µm, 50µm, 5µm) Quantification of tdTom positive cells within each vessel lumen size, expressed as a percentage of total lumen cells. **(C)** IF staining for tdTom and αSMA in control and Lats1/2-KO D20 lungs (large scale bar 500µm, zoom-in scale bar 100µm) and quantification of normalized area covered by αSMA (n= 6 control/ 5 knockout mice per group), analyzed using a one-way ANOVA. **(D)** IF staining for Col1a1 in control and Lats1/2-KO D10, D15 and D20 lungs (scale bar 20µm). **(E)** Picrosirius Red staining of control and Lats1/2-KO mice at D10, D15, and D20 (scale bar 50 µm). **(F)** Evans blue quantification from whole lung lysates (n= 4 mice per group) analyzed using a two-tailed Student's t-test, and representative picture of control and Lats1/2-KO D20 lungs 30 minutes after Evans Blue administration. **(G)** IF staining for CDH5, Actin (Phalloidin) in Human Lung Microvascular Endothelial cells (HLMECs) treated with control siRNA or siRNA targeting LATS1/2 (scale bar 50µm). **(H)** Trans-endothelial electrical resistance (TEER) measurements across two HLMECs lines treated with control siRNA or siRNA targeting LATS1/2 (n=3 wells per condition). Statistical significance using a two-tailed Student's t-test. **(I)** Two HLMECs lines treated with control siRNA or siRNA targeting LATS1/2 and grown on a transwell insert and then fluorescently conjugated dextran was administered on the apical compartment. The media from the basal compartment was isolated 60 minutes later and analyzed on a spectrometer for florescence leak. Statistical significance was assessed using a Two-tailed Student's t-test (n=3 wells per condition).

Fig S2

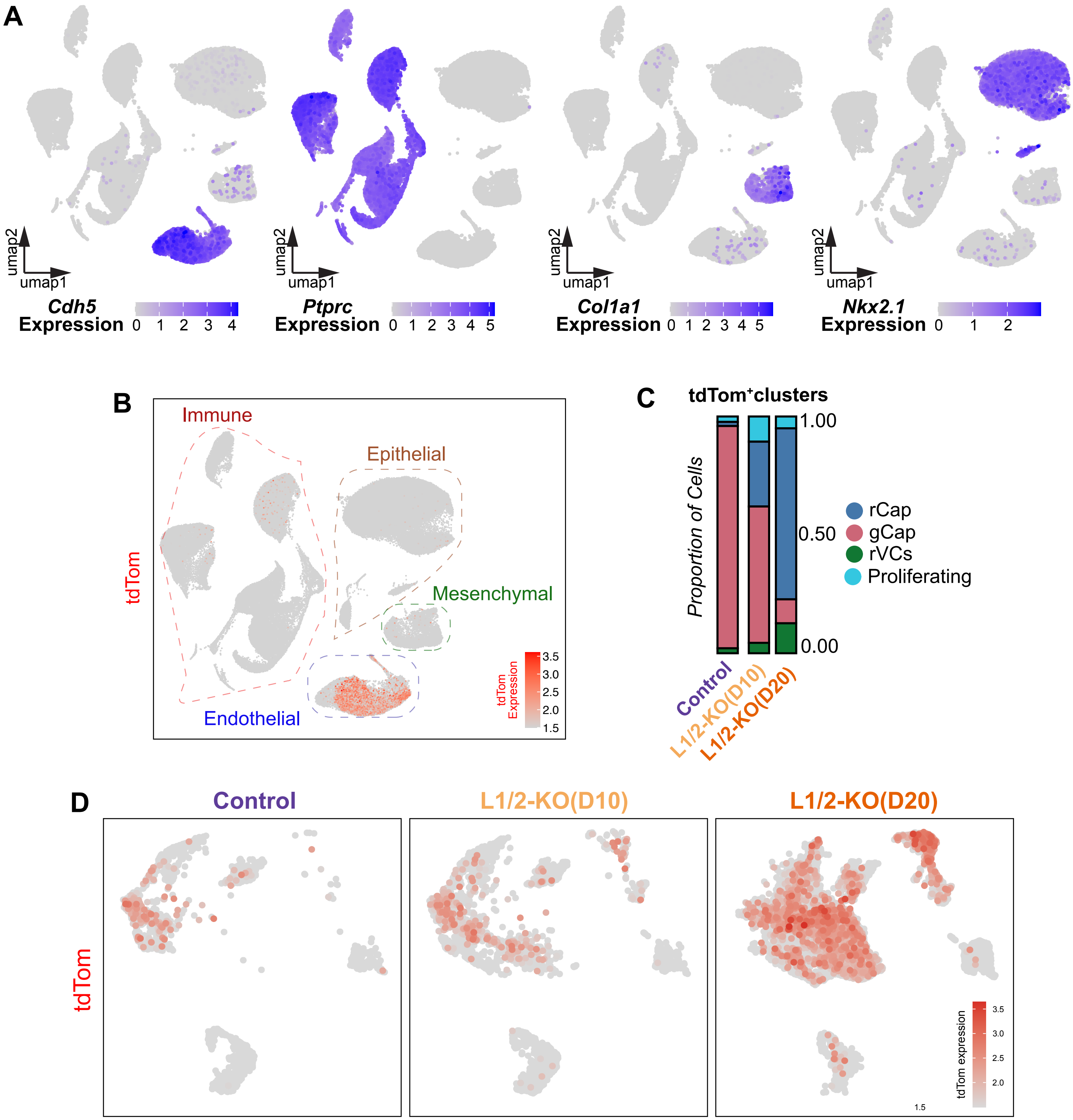
A
umap2
umap1
Cdh5 Expression
0 1 2 3 4
Ptprc Expression
0 1 2 3 4 5
Col1a1 Expression
0 1 2 3 4 5
Nkx2.1 Expression
0 1 2
B
Immune
Epithelial
Mesenchymal
Endothelial
tdTom
tdTom Expression
3.5
3.0
2.5
2.0
1.5
C
tdTom+clusters
Proportion of Cells
1.00
0.50
0.00
rCap
gCap
rVCs
Proliferating
Control
L1/2-KO(D10)
L1/2-KO(D20)
D
Control
L1/2-KO(D10)
L1/2-KO(D20)
tdTom
tdTom expression
3.5
3.0
2.5
2.0
1.5

<u>Fig S2</u>**.** **(A)** UMAP projection of *Cdh5, Ptprc, Col1a1* and *Nkx2.1* expression across all cells identified from the scRNA-seq analysis of Control and Lats1/2-KO lungs. **(B)** UMAP projection of tdTom expression across all cells identified in the scRNA-seq experiment. **(C)** Cell proportion analysis of $tdTom^+$ ECs across Control and Lats1/2-KO timepoints lungs per timepoint. **(D)** UMAP projection of tdTom expression in all ECs identified from the scRNA-seq analysis of Control and Lats1/2-KO lungs split per timepoint.

Fig S3

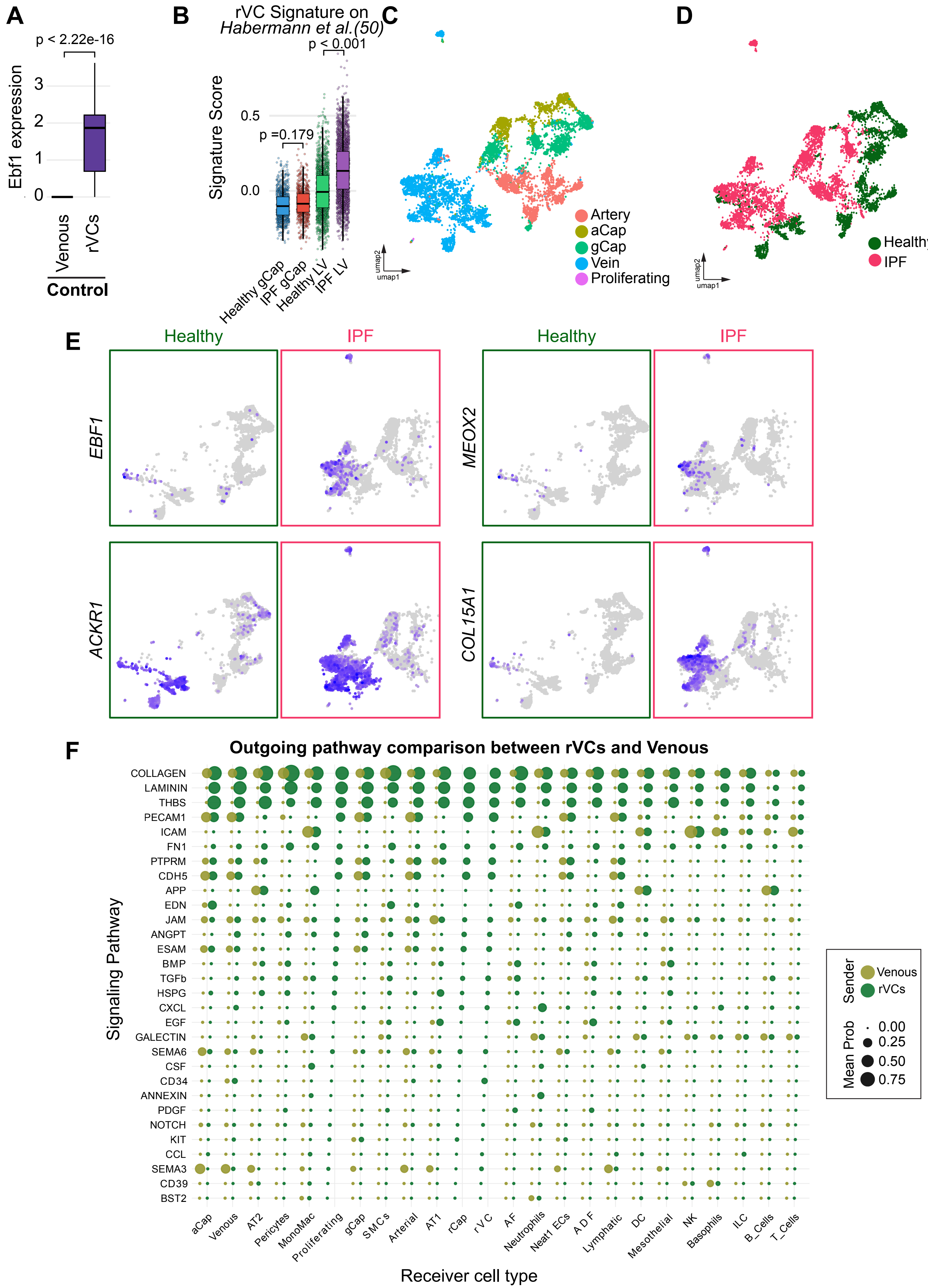
A
p < 2.22e-16
Ebf1 expression
Venous
rVCs
Control
B
rVC Signature on
Habermann et al.(50)
p < 0.001
p =0.179
Signature Score
Healthy gCap
IPF gCap
Healthy LV
IPF LV
C
Artery
aCap
gCap
Vein
Proliferating
umap1
umap2
D
Healthy
IPF
E
Healthy
IPF
EBF1
MEOX2
ACKR1
COL15A1
F
Outgoing pathway comparison between rVCs and Venous
Signaling Pathway
COLLAGEN
LAMININ
THBS
PECAM1
ICAM
FN1
PTPRM
CDH5
APP
EDN
JAM
ANGPT
ESAM
BMP
TGFb
HSPG
CXCL
EGF
GALECTIN
SEMA6
CSF
CD34
ANNEXIN
PDGF
NOTCH
KIT
CCL
SEMA3
CD39
BST2
aCap
Venous
AT2
Pericytes
MonoMac
Proliferating
gCap
SMCs
Arterial
AT1
rCap
rVC
AF
Neutrophils
Neat1 ECs
ADF
Lymphatic
DC
Mesothelial
NK
Basophils
ILC
B_Cells
T_Cells
Receiver cell type
Sender
Venous
rVCs
Mean Prob
0.00
0.25
0.50
0.75

***Fig S3*.** **(A)** Box plot depicting averaged *Ebf1* expression across venous and rVCs identified by scRNA-seq in Control lungs. Statistical significance was assessed by performing a two-sided unpaired Wilcoxon rank sum-test. **(B)** Lats1/2-KO-rVC gene signature enrichment on the *Habermann et al.* (50) dataset. Statistical significance was assessed using a non-parametric two-way aligned rank transform ANOVA followed by Benjamini-Hochberg-adjusted post hoc contrasts. **(C)** UMAP projection of all ECs identified in the *Habermann et al.* (50) dataset (n=6,264 cells). **(D)** UMAP projection of all ECs split per disease grouping in the *Habermann et al.* (50) dataset. **(E)** UMAP projection depicting gene expression for the following markers: *EBF1, MEOX2, ACKR1* and *COL15A1* in the *Habermann et al.* (50) dataset. **(F)** Bubble plot depicting CellChat analysis comparing Lats1/2-KO (D10 and D20 combined) rVCs (green) and Control venous EC(olive green) outgoing pathway signaling to all cell types. Dot size corresponds to signaling probability.

Fig S4

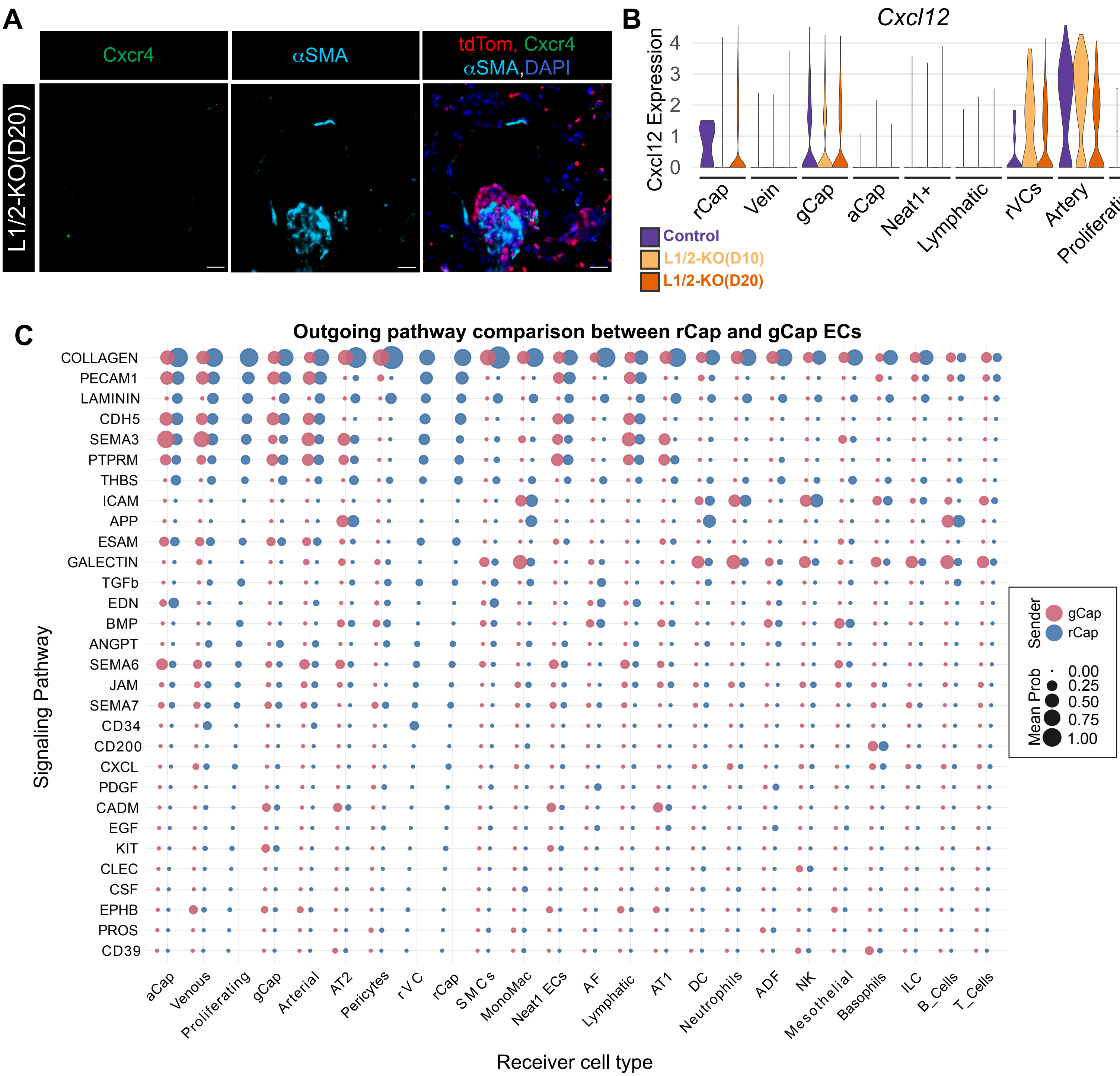
A
Cxcr4
αSMA
tdTom, Cxcr4
αSMA,DAPI
L1/2-KO(D20)
B
Cxcl12
Cxcl12 Expression
0
1
2
3
4
rCap
Vein
gCap
aCap
Neat1+
Lymphatic
rVCs
Artery
Proliferating
Control
L1/2-KO(D10)
L1/2-KO(D20)
C
Outgoing pathway comparison between rCap and gCap ECs
Signaling Pathway
COLLAGEN
PECAM1
LAMININ
CDH5
SEMA3
PTPRM
THBS
ICAM
APP
ESAM
GALECTIN
TGFb
EDN
BMP
ANGPT
SEMA6
JAM
SEMA7
CD34
CD200
CXCL
PDGF
CADM
EGF
KIT
CLEC
CSF
EPHB
PROS
CD39
aCap
Venous
Proliferating
gCap
Arterial
AT2
Pericytes
rVC
rCap
SMCs
MonoMac
Neat1 ECs
AF
Lymphatic
AT1
DC
Neutrophils
ADF
NK
Mesothelial
Basophils
ILC
B_Cells
T_Cells
Receiver cell type
Sender
gCap
rCap
Mean Prob
0.00
0.25
0.50
0.75
1.00

Fig S4. **(A)** IF staining for tdTom, αSMA and Cxcr4 in Lats1/2-KO D20 lungs (scale bar 20μm). **(B)** Violin plot depicting expression of *Cxcl12* expression split across control and Lats1/2-KO timepoints for each EC subpopulation. **(C)** Bubble plot depicting CellChat analysis comparing Lats1/2-KO (D10 and D20 combined) rCap (blue) and Control gCap EC(light red) outgoing pathway signaling to all cell types. Dot size corresponds to signaling probability.

Fig S5

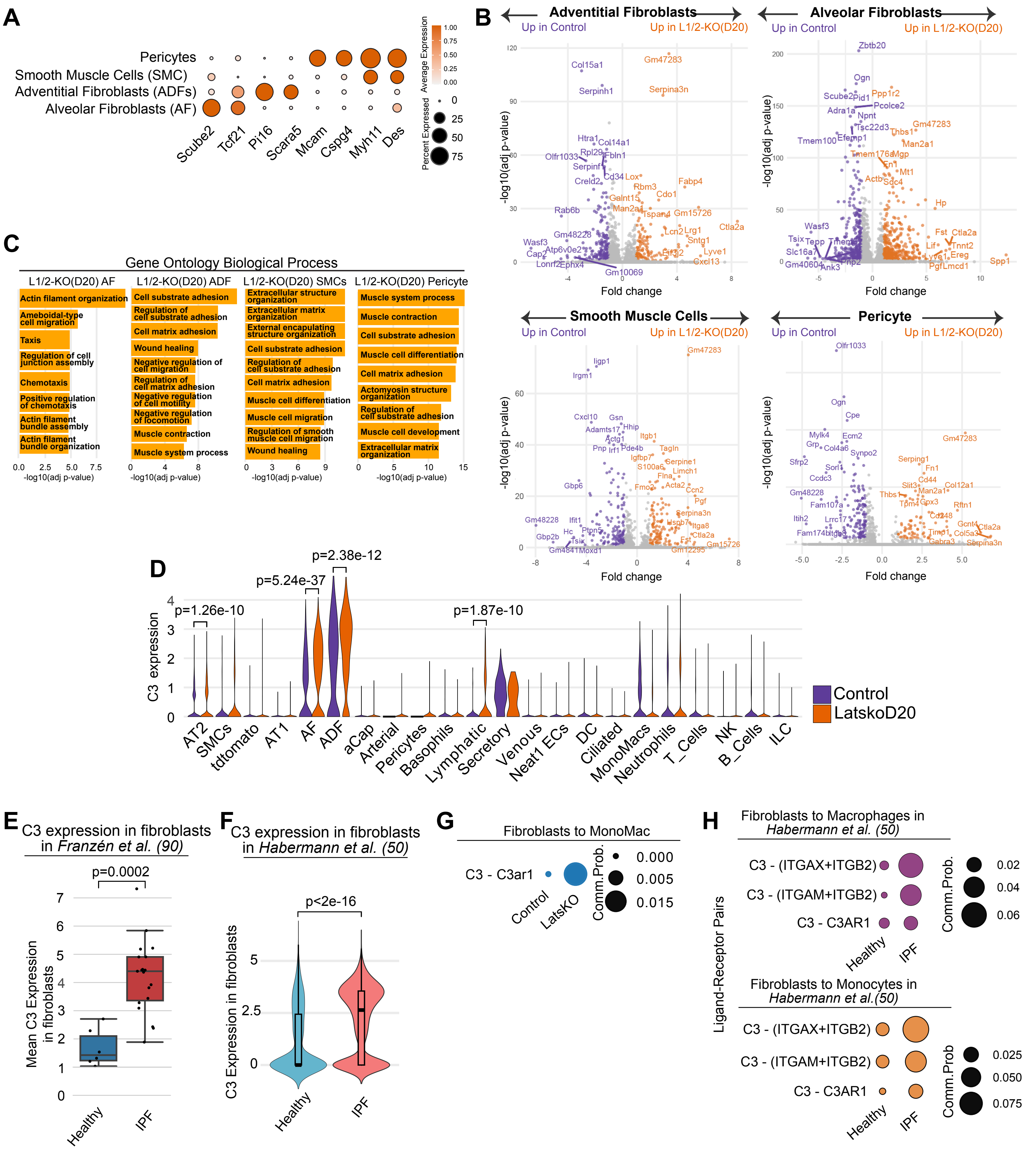
A
Pericytes
Smooth Muscle Cells (SMC)
Adventitial Fibroblasts (ADFs)
Alveolar Fibroblasts (AF)
Scube2
Tcf21
Pi16
Scara5
Mcam
Cspg4
Myh11
Des
Average Expression
Percent Expressed
B
Adventitial Fibroblasts
Alveolar Fibroblasts
Smooth Muscle Cells
Pericyte
Up in Control
Up in L1/2-KO(D20)
-log10(adj p-value)
Fold change
C
Gene Ontology Biological Process
L1/2-KO(D20) AF
L1/2-KO(D20) ADF
L1/2-KO(D20) SMCs
L1/2-KO(D20) Pericyte
-log10(adj p-value)
D
C3 expression
p=1.26e-10
p=5.24e-37
p=2.38e-12
p=1.87e-10
Control
LatskoD20
E
C3 expression in fibroblasts in Franzén et al. (90)
p=0.0002
Mean C3 Expression in fibroblasts
Healthy
IPF
F
C3 expression in fibroblasts in Habermann et al. (50)
p<2e-16
C3 Expression in fibroblasts
Healthy
IPF
G
Fibroblasts to MonoMac
C3 - C3ar1
Control
LatsKO
Comm.Prob.
H
Fibroblasts to Macrophages in Habermann et al. (50)
C3 - (ITGAX+ITGB2)
C3 - (ITGAM+ITGB2)
C3 - C3AR1
Healthy
IPF
Fibroblasts to Monocytes in Habermann et al.(50)
Ligand-Receptor Pairs
Comm.Prob

Fig S5**.** **(A)** Bubble plot with lung fibroblast subtype specific markers. **(B)** Volcano plots comparing differential expressed genes in control and Lats1/2-KO D20 mesenchymal subpopulations. Differential expression significance was determined using Benjamini-Hochberg-adjusted p-values. **(C)** GO analysis on Lats1/2-KO D20 AFs, ADFs, SMCs and Pericytes. **(D)**Violin plot of C3 expression for all cell types identified in the scRNA-seq, split between control and Lats1/2-KO D20, with statistical significance calculated with a Wilcoxon rank-sum test. **(E)** Box plot depicting C3 expression in human fibroblasts split between healthy and IPF samples in *Franzén et al* (90)*,* with statistical significance evaluate with a non-parametric Wilcoxon rank-sum test. **(F)** Violin plot depicting C3 expression in human fibroblasts split between healthy and IPF samples in *Habermann et al.* (50), with statistical significance evaluated using a Wilcoxon rank-sum test. **(G)** Bubble plot showing CellChat analysis of C3-C3ar1 communication probability between fibroblasts (sender) and macrophage/monocytes (receiver) in Lats1/2-KO (D10 and D20 combined) and Control lungs. **(H)** Bubble plots depicting CellChat analysis of ligand-receptor pair communication probability between human fibroblasts (sender) and macrophages (receiver)(top) or monocytes (receiver)(bottom) in healthy and IPF lungs from *Habermann et al.* (50). For all CellChat analysis, interaction probabilities were summed across targets, normalized and ranked, with dot size indicating scaled communication strength.

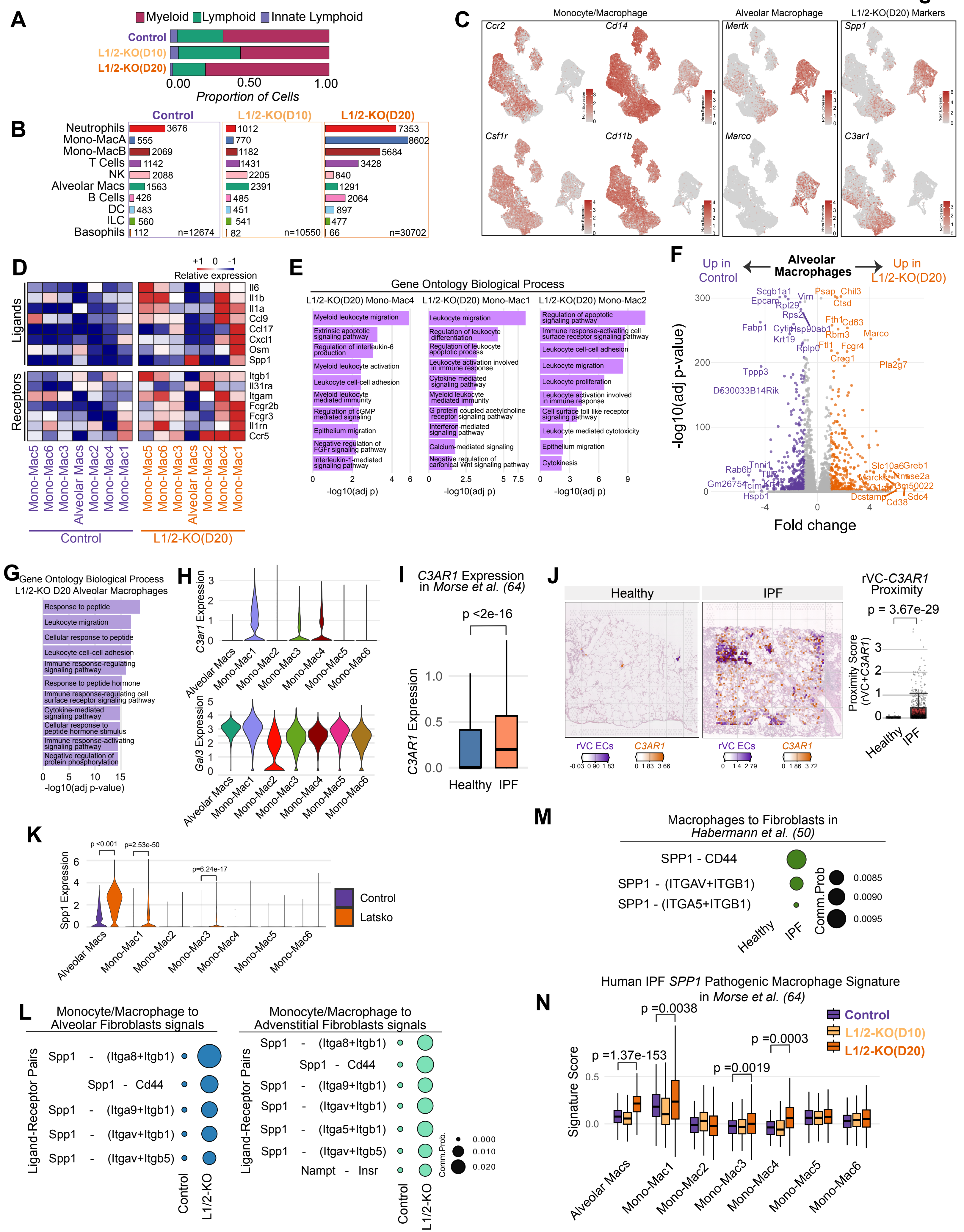
A
Myeloid
Lymphoid
Innate Lymphoid
Control
L1/2-KO(D10)
L1/2-KO(D20)
Proportion of Cells
B
Neutrophils
Mono-MacA
Mono-MacB
T Cells
NK
Alveolar Macs
B Cells
DC
ILC
Basophils
n=12674
n=10550
n=30702
C
Monocyte/Macrophage
Alveolar Macrophage
L1/2-KO(D20) Markers
D
Relative expression
Ligands
Receptors
Control
L1/2-KO(D20)
E
Gene Ontology Biological Process
L1/2-KO(D20) Mono-Mac4
L1/2-KO(D20) Mono-Mac1
L1/2-KO(D20) Mono-Mac2
-log10(adj p)
F
Up in Control
Alveolar Macrophages
Up in L1/2-KO(D20)
-log10(adj p-value)
Fold change
G
Gene Ontology Biological Process
L1/2-KO D20 Alveolar Macrophages
-log10(adj p-value)
H
Csar1 Expression
Gal3 Expression
I
C3AR1 Expression in Morse et al. (64)
p <2e-16
C3AR1 Expression
Healthy
IPF
J
Healthy
IPF
rVC-C3AR1 Proximity
p = 3.67e-29
Proximity Score (rVC+C3AR1)
rVC ECs
C3AR1
K
p <0.001
p=2.53e-50
p=6.24e-17
Spp1 Expression
Control
Latsko
L
Monocyte/Macrophage to Alveolar Fibroblasts signals
Monocyte/Macrophage to Adventstitial Fibroblasts signals
Ligand-Receptor Pairs
Control
L1/2-KO
Comm.Prob.
M
Macrophages to Fibroblasts in Habermann et al. (50)
SPP1 - CD44
SPP1 - (ITGAV+ITGB1)
SPP1 - (ITGA5+ITGB1)
Comm.Prob
Healthy
IPF
N
Human IPF SPP1 Pathogenic Macrophage Signature in Morse et al. (64)
p =1.37e-153
p =0.0038
p =0.0019
p =0.0003
Control
L1/2-KO(D10)
L1/2-KO(D20)
Signature Score
Alveolar Macs
Mono-Mac1
Mono-Mac2
Mono-Mac3
Mono-Mac4
Mono-Mac5
Mono-Mac6

Fig S6**.** **(A)** Cell proportion analysis of myeloid, lymphoid and innate lymphoid cell populations identified in scRNA-seq analysis of Control and D10 or D20 Lats1/2-KO lungs. **(B)** Immune cell numbers identified across the different scRNA-seq conditions. **(C)** UMAP projection of *Ccr2, Csf1r, Cd11b, Cd14, Marco, Mertk*, *Spp1* and *C3ar1* expression across macrophage/monocytes identified in scRNA-seq. **(D)** Heatmap depicting Ligand and Receptor genes distinctly induced in Lats1/2-KO D20 macrophages/monocytes. **(E)** GO enrichment of genes expressed by Lats1/2-KO D20 Mono-Mac1, Mono-Mac2 and Mono-Mac4 clusters**.** **(F)** Volcano plot comparing differentially expressed genes between control and Lats1/2-KO D20 alveolar macrophages. Significance was determined using Benjamini-Hochberg-adjusted p-values. **(G)** Gene Ontology enrichment analysis of genes induced in Lats1/2-KO D20 alveolar macrophages**.** **(H)** Violin plots depicting *C3ar1* and *Gal3* expression across all macrophage/monocytes from Control and Lats1/2-KO lungs. **(I)** *C3AR1* expression in human macrophages from healthy and IPF lungs in *Morse et al.* (64). Significance was assessed using two-sided Wilcoxon rank-sum tests. **(J)** Spatial mapping of the Lats1/2-KO-rVC gene signature (purple) and *C3AR1* expression (orange) across a healthy and IPF sample from *Franzén et al* (90) (left). Quantification of spatial proximity between the Lats1/2-KO-rVC gene signature and *C3AR1* expression through a patch-based neighborhood analysis (right). Significance was evaluated with a non-parametric Wilcoxon rank-sum test. **(K)** Violin plot of *Spp1* expression across all macrophage/ monocyte subpopulations split between Control and Lats1/2-KO (D10 and D20 combined), with significance calculate with a Wilcoxon rank-sum test. **(L)** CellChat analysis of *Spp1* communication probability between macrophage/monocytes (sender) and alveolar (receiver)(left) or adventitial fibroblasts (receiver)(right) in Lats1/2-KO (D10 and D20 combined) and Control lungs. **(M)** Bubble plots depicting CellChat analysis of ligand-receptor pair communication probability between human macrophages (sender) and fibroblasts (receiver) in healthy and IPF from *Habermann et al.* (50). **(N)** Enrichment analysis of *SPP1*+ macrophage gene signature from human IPF (*Morse et al.* (64)) on clusters from control and Lats1/2-KO lungs split per timepoint. Significance was assessed using a two-sided Kruskal-Wallis rank-sum test, followed by Dunn's post-hoc test with Benjamini-Hochberg FDR correction.

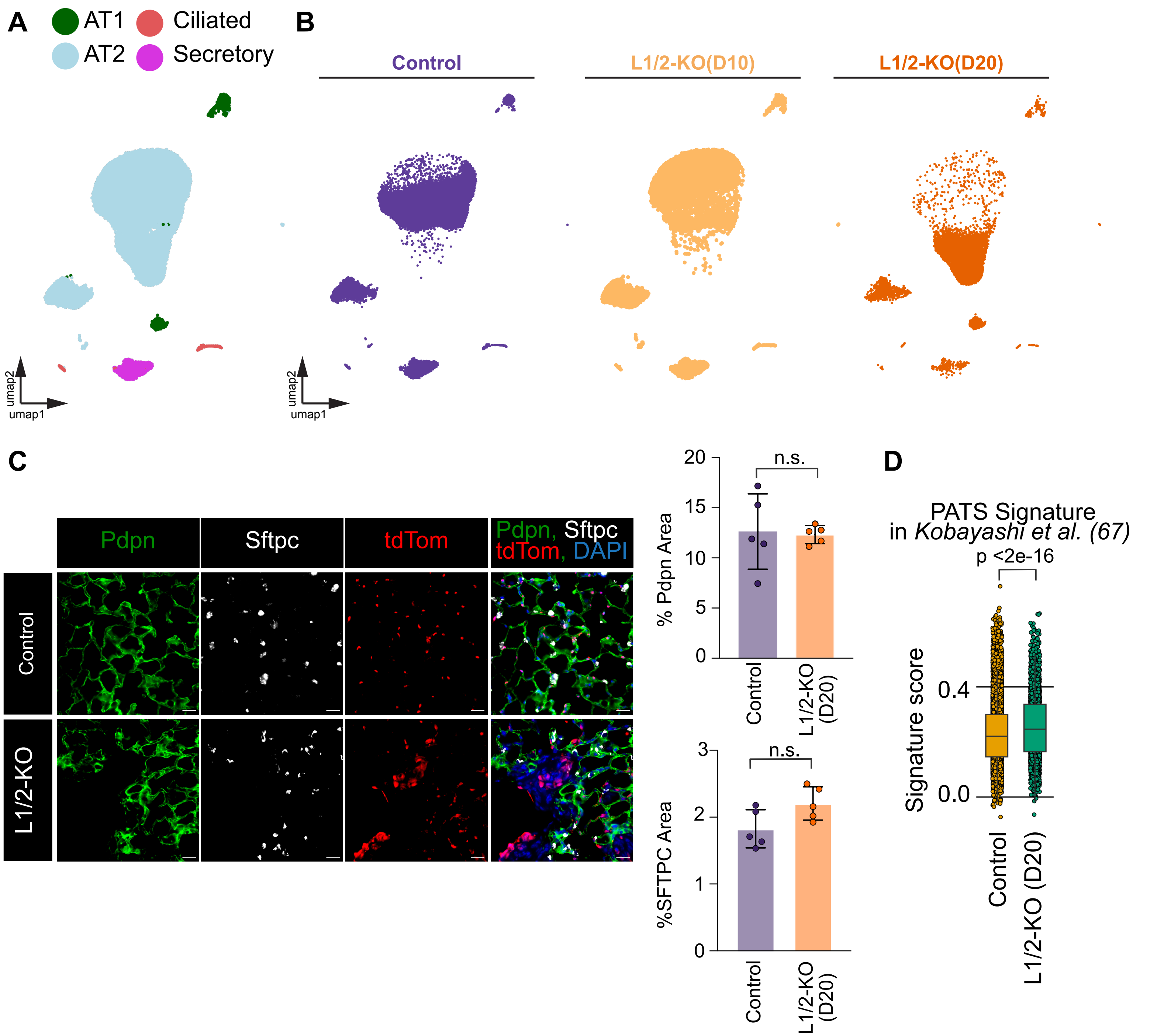
A
AT1
Ciliated
AT2
Secretory
umap2
umap1
B
Control
L1/2-KO(D10)
L1/2-KO(D20)
umap2
umap1
C
Pdpn
Sftpc
tdTom
Pdpn, Sftpc
tdTom, DAPI
Control
L1/2-KO
% Pdpn Area
20
15
10
5
0
n.s.
Control
L1/2-KO
(D20)
%SFTPC Area
3
2
1
0
n.s.
Control
L1/2-KO
(D20)
D
PATS Signature
in Kobayashi et al. (67)
p <2e-16
Signature score
0.4
0.0
Control
L1/2-KO (D20)

Fig S7**.** **(A)** UMAP projection of unbiased clustering of all epithelial cells (n= 58,722) identified in the scRNA-seq analysis of control and Lats1/2-KO lungs. **(B)** UMAP projection of all epithelial cells split per timepoint. **(C)** IF analysis of Sftpc, Pdpn and tdTom in Control and Lats1/2-KO(D20) lungs, along with associated quantitation (n=5 Control and Lats1/2-KO D20 lungs). Statistical significance determined using a Two-tailed Student's t-test **(D)** Gene signature enrichment of the Pre alveolar type-1 transitional cell state (PATS) gene list from the *Kobayashi et al.* (67) dataset on control and Lats1/2-KO D20 AT2 cells. Statistical significance was assessed using two-sided Wilcoxon rank-sum tests comparing pathway probability between conditions, followed by Benjamini-Hochberg false discovery rate (FDR) correction across pathways.

Fig S8

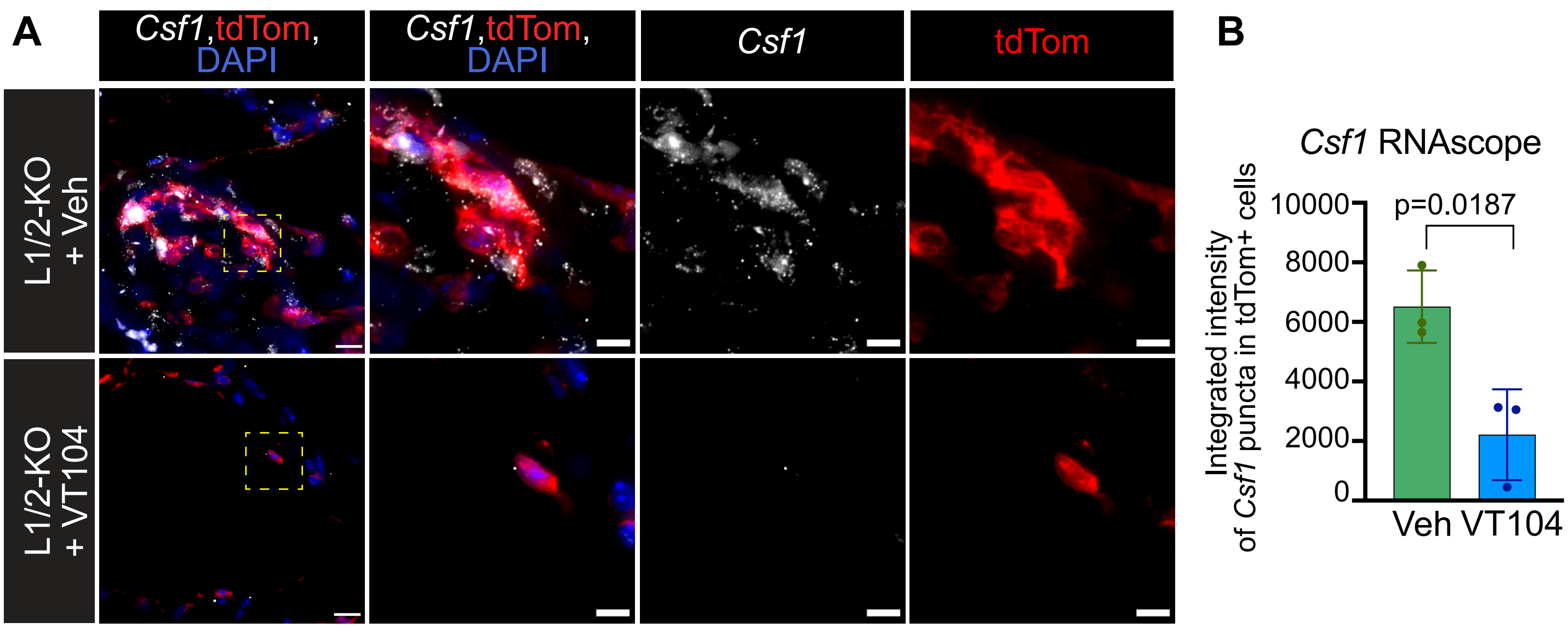
A
Csf1,tdTom,
DAPI
Csf1,tdTom,
DAPI
Csf1
tdTom
L1/2-KO
+ Veh
L1/2-KO
+ VT104
B
Csf1 RNAscope
Integrated intensity
of Csf1 puncta in tdTom+ cells
10000
8000
6000
4000
2000
0
p=0.0187
Veh
VT104

Fig S8. **(A)** IF for tdTom and *Csf1* RNAscope on vehicle and VT104-treated Lats1/2-KO-D20 lungs (large scale bar 10µm, zoom-in scale bar 5µm) (n=3 per group). **(B)** Quantification of integrated *Csf1* puncta intensity on vehicle and VT104-treated Lats1/2-KO-D20 lungs (n=3 per group). Values are summarized as mean +/- SEM and a two-tailed Student's t-test was performed.

SUPPLEMENTARY TABLES

Table S1**.** Fibrosis and Hippo related genes, bubble plot statistics across rVCs/rCaps, rVC Heatmap and rCap Heatmap (related to Fig2H).

Table S2**.** Top distinctly enriched genes expressed in rVCs (related to Fig3B).

Table S3**.** Gene Signatures used for enrichment analyses.

Table S4**.** CellChat comparison of outgoing signals in rVCs vs Veins.

Table S5**.** Top distinctly enriched genes expressed in rCaps (related to Fig4A).

Table S6**.** CellChat comparison of outgoing signals in rCap versus gCap ECs.

Table S7**.** Fibroblast identity marker and Control-L1/2-KO comparison bubble plots with statistics (related to Fig S5A & Fig5B).

Table S8**.** Differentially expressed genes from Fibroblast volcano plots comparing Control and L1/2KO (related to FigS5B).

Table S9**.** Mono-macs heatmaps (related to Fig6G and FigS6D).

Table S10**.** Alveolar macrophage volcano plot comparing Control and L1/2KO (related to FigS6F).

Table S11**.** Genes from AT2 volcano plot comparing Control and L1/2KO (related to Fig 7D).

Table S12**.** AT2 injury-associated markers bubble plot statistics (related to Fig7F).

Table S13**.** Antibodies used in the study.

Table S14**.** Computational tools used in the study.
